# Metabolism of sucrose in a non-fermentative *Escherichia coli* under oxygen limitation

**DOI:** 10.1007/s00253-019-09909-6

**Published:** 2019-05-31

**Authors:** Karel Olavarria, Albert Fina, Mariana I. Velasco, Mark C. M. van Loosdrecht, Sebastian Aljoscha Wahl

**Affiliations:** Department of Biotechnology, Applied Sciences Faculty, Delft University of Technology, van der Maasweg 9, 2629 HZ Delft, The Netherlands

**Keywords:** Sucrose phosphorolysis, Flux balance analysis, Plasmid burden, Data reconciliation, Carbon storage regulator A

## Abstract

Biotechnological industry strives to develop anaerobic bioprocesses fueled by abundant and cheap carbon sources, like sucrose. However, oxygen-limiting conditions often lead to by-product formation and reduced ATP yields. While by-product formation is typically decreased by gene deletion, the breakdown of oligosaccharides with inorganic phosphate instead of water could increment the ATP yield. To observe the effect of oxygen limitation during sucrose consumption, a non-fermentative *Escherichia coli* K-12 strain was transformed with genes enabling sucrose assimilation. It was observed that the combined deletion of the genes *adhE*, *adhP*, *mhpF*, *ldhA*, and *pta* abolished the anaerobic growth using sucrose. Therefore, the biomass-specific conversion rates were obtained using oxygen-limited continuous cultures. Strains performing the breakdown of the sucrose by hydrolysis (SUC-HYD) or phosphorolysis (SUC-PHOSP) were studied in such conditions. An experimentally validated in silico model, modified to account for plasmid and protein burdens, was employed to calculate carbon and electron consistent conversion rates. In both strains, the biomass yields were lower than expected and, strikingly, SUC-PHOSP showed a yield lower than SUC-HYD. Flux balance analyses indicated a significant increase in the non-growth-associated ATP expenses by comparison with the growth on glucose. The observed fructose-1,6-biphosphatase and phosphoglucomutase activities, as well as the concentrations of glycogen, suggest the operation of ATP futile cycles triggered by a combination of the oxygen limitation and the metabolites released during the sucrose breakdown.

**Electronic supplementary material:**

The online version of this article (10.1007/s00253-019-09909-6) contains supplementary material, which is available to authorized users.

## Introduction

The design of efficient and sustainable biotechnological processes demands the maximization of product titer, production rate, and yield, and the reduction of fixed and variable costs (Porro et al. 2014). All these demands call for the development of anaerobic processes as opposed to aerobic cultivations (Cueto-Rojas et al. 2015). Further reduction of variable costs is obtained when using abundant, cheap feedstocks like sucrose (Marques et al. 2016).

Although *E. coli* K-12–derived strains can anaerobically grow using sucrose as the sole carbon source (Hoefel et al. 2012), the bio-energetic impact of the oxygen limitation while using this carbon source has not been studied in detail. This kind of analysis is required to improve the efficiency of the bioprocesses fueled by sucrose.

On the other hand, despite the advantages of the anaerobic conversions, the generation of by-products and the decrease in the ATP yield under such conditions often represent important obstacles. Some of the by-products generated by *E. coli* are weak acids. As well as diverting carbon otherwise available for the generation of the target product(s), weak acids could be transported cyclically across the membrane, leading to ATP losses (Axe and Bailey 1995). Moreover, the presence of such by-products could trigger physiological responses whose effects might be difficult to anticipate and quantify (Roe et al. 2002). Therefore, the suppression of these by-products is usually beneficial for the bioprocesses, and gene deletion is the tool typically used to achieve this goal (Jantama et al. 2008; Jian et al. 2010; Wang et al. 2012). However, the consequences of such deletions on the redox-balancing mechanisms need to be considered.

Regarding the decrease in ATP yield, it is important to highlight that this could make the generation of some biotechnologically relevant products unfeasible. One of the strategies employed to increase the ATP yield is the use of phosphorylases (also known as phosphorolases) to catalyze the breakdown of oligosaccharides (de Kok et al. 2011; Marques et al. 2018). The incorporation of inorganic phosphate during the breakdown of the glycosidic bonds means a saving in the ATP consumed to phosphorylate the monosaccharides fueling the glycolysis (Zhang and Lynd 2005). Therefore, in addition to its abundance and low price, using sucrose as substrate has the potential to enhance the ATP yield during (anaerobic) fermentations in *E. coli*.

Different oligosaccharide phosphorylases have been found (Mukherjee et al. 2018) or have been heterologously expressed in *E. coli*, including the sucrose phosphorylase from *Bifidobacterium adolescentis* (De Bruyn et al. 2015). Nevertheless, to the best of our knowledge, there are no published studies about the impact on the biomass yield of implementing oligosaccharide phosphorolysis in *E. coli*.

**Fig. 1:**
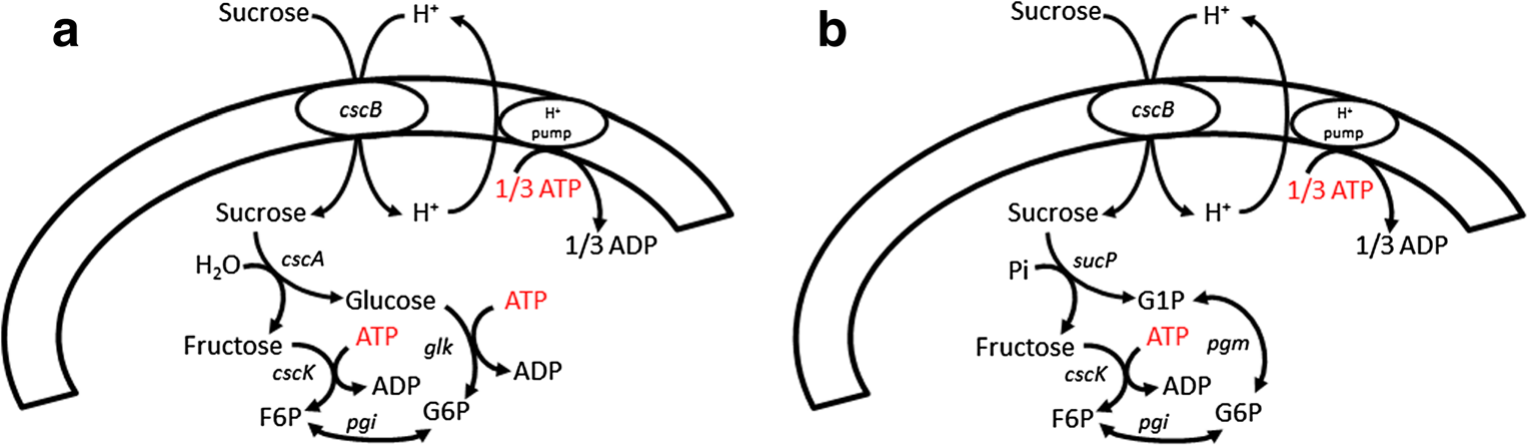
Functions of the proteins encoded by the genes *cscA*, *cscB*, *cscK*, and *sucP*. The differences in the spent ATP to obtain fructose-6-phosphate depending on the mechanism to break down the sucrose (water in **a**, phosphate in **b**) are also highlighted. Some enzymes and the sucrose/H^+^ symporter are represented with the names of the corresponding encoding genes. F6P, fructose-6-phosphate; G6P, glucose-6-phosphate; G1P, glucose-1-phosphate; Pi, inorganic phosphate

The aim of this study was the quantitative analysis of the cellular energetics, especially biomass yield, of *E. coli* K-12 under conditions relevant for the biotechnological industry: using a cheap and abundant carbon source (sucrose), with a limited oxygen supply, and using genetically modified cells to diminish by-product generation. Having this objective, we expressed genes enabling the sucrose uptake and hydrolysis/phosphorolysis (Fig. 1) in a mutant *E. coli* K-12 with deletions reducing its capacity to generate by-products such as ethanol, acetate, lactate, and formate. The biomass-specific conversion rates of mutant strains breaking down the sucrose using water or inorganic phosphate were compared.

## Materials and methods

### Construction of the strains

The characteristics of the hosting strains are summarized in the Table 1. The hosting strain TUD58 has a DE3 insertion on the *lacZ* locus (Tseng et al. 2009) and deletions of the genes *adhE* (encoding for an alcohol dehydrogenase), *adhP* (encoding for another alcohol dehydrogenase), *ldhA* (encoding for the lactate dehydrogenase), *pta* (encoding for the phosphotransacetylase), and *mhpF* (encoding for an acetaldehyde dehydrogenase). These deletions had already been performed, using the method described by Wanner and Datsenko (Datsenko and Wanner 2000). The resulting strain after these deletions was kindly donated by Ana Sofia Araújo Ferreira and Isabel Rocha from University of Minho, Portugal. Details of the construction of the plasmids pUC19-*cscKB*, pUC19-csc*AKB*, and pUC19-*cscKB*-*sucP* are described in Supplementary Material 1. The sequences of the employed primers are listed in Table S1. The supplementary files *pUC19-cscKB.gb*, *pUC19-cscAKB.gb*, and *pUC19-cscKB-sucP.gb* contain the DNA sequences of these plasmids. These plasmids were introduced, by electroporation, into cells of the strains *E. coli* K-12 MG1655 and TUD58.

**Table 1:** *E. coli* K-12 strains employed in this study

Strain	Background	Plasmid
MG1655	(F–λ–*ilvG*–*rfb*-50 *rph*-1) DSM 498, ATCC 23716, CECT 7619, CIP 110067	–
TUD58	(F–λ–*ilvG*–*rfb*-50 *rph*-1 (DE3)) Δ*adhE* Δ*adhP* Δ*ldhA* Δ*pta* Δ*mhpF*	–
TUD37	(F–λ–*ilvG*–*rfb*-50 *rph*-1)	pUC19-*cscBK*
TUD66	(F–λ–*ilvG*–*rfb*-50 *rph*-1)	pUC19-*cscAKB*
TUD69	(F–λ–*ilvG*–*rfb*-50 *rph*-1)	pUC19-*cscKB*-*sucP*
TUD38	(F–λ–*ilvG*–*rfb*-50 *rph*-1 (DE3)) Δ*adhE* Δ*adhP* Δ*ldhA* Δ*pta* Δ*mhpF*	pUC19-*cscBK*
SUC-HYD	(F–λ–*ilvG*–*rfb*-50 *rph*-1 (DE3)) Δ*adhE* Δ*adhP* Δ*ldhA* Δ*pta* Δ*mhpF*	pUC19-*cscAKB*
SUC-PHOSP	(F–λ–*ilvG*–*rfb*-50 *rph*-1 (DE3)) Δ*adhE* Δ*adhP* Δ*ldhA* Δ*pta* Δ*mhpF*	pUC19-*cscKB*-*sucP*

### Cultures in batch

Mineral medium supplemented with 15 g/L of sucrose or 15 g/L of glucose was used for the cultures in batch. The content of one liter of mineral medium (excluding the carbon source) was 5 g of (NH_4_)SO_4_, 2 g KH_2_PO_4_, 0.5 g MgSO_4_·7H_2_O, 0.5 g NaCl, 2 g NH_4_Cl, 0.001 g of thiamine, and 2 mL of a trace-element solution prepared as previously described (Verduyn et al. 1992). All the components of the medium, excluding the carbon source, the thiamine, and the trace elements, were mixed and the pH of the medium was adjusted to 7.0 using a solution of 2 M KOH before sterilization in autoclave. After autoclaving, the thiamine, the trace elements, and the carbon source were added to the sterile media using a syringe coupled to a 0.22-μm polyvinyl difluoride (PVDF) filter (Millipore, Germany). All the experiments in batch were performed in triplicate.

For the growth experiments under anaerobic conditions, the medium was supplemented with 0.24 mg/L of Na_2_SeO_3_ × 5 H_2_O, 0.28 mg/L Na_2_MoO_4_, and 2.3 mg/L Ni(NO_3_)_2_ × 6 H_2_O to provide the metals required for the activity of the formate hydrogen lyase (Soini et al. 2008), and it was also supplemented with 50 mM of 3-(N-morpholino)propanesulfonic acid (MOPS) to increase the buffering capacity of the medium. For these experiments, sealed bottles were used. Once the medium was inside the sealed bottles, a continuous stream of sterile compressed nitrogen was sparged into the bottles to remove the oxygen from both the liquid and the headspace. The sealed bottles were inoculated with cells taken from an aerobic pre-inoculum culture grown on the same medium. The initial optical density of the cultures at 600 nm was 0.1.

### Continuous cultures

The growth medium employed for the continuous cultures was the same as that employed for the cultures in batch. The continuous cultures were performed at dilution rates of 0.05 h^−1^ and the aeration rate was kept constant at 0.55 v.v.m. The stirring speed was initially set to 500 r.p.m. to achieve a steady state in fully aerobic conditions (dissolved oxygen tension above 50%) (Supplementary Material 2, Table S2, Fig. S2 and supplementary file *calculating required stirring.xls*).

The stirring rate was gradually decreased to achieve the oxygen limitation. The signals of dissolved oxygen tension and CO_2_ in the outgas were followed until non-stable fluctuations in CO_2_ in the outgas were observed. Once the oxygen-limited steady states were approached, samples of broth, biomass, and cell-free extracellular media (filtrates) were obtained at two different times during the same steady state. Continuous cultures were assumed to be in steady state when the signal corresponding to the CO_2_ in the outgas remained stable for at least two culture volume changes. Other details of the setup, operation, sampling, and sample analyses of the continuous cultures are explained in Supplementary Materials 3 and 4.

### Enzymatic assays

Cells harvested from batch cultures during the exponential growth phase or from the continuous cultures were used for determination of the specific activities. Three different assays were performed: sucrose phosphorylase (SucP), phosphoglucomutase, and fructose-1,6-bisphosphatase. These three experiments were coupled assays leading to the production of NADPH from NADP^+^. Thus, the production of NADPH, which was followed spectrophotometrically at 340 nm, was used to calculate the reaction rate. All the enzymatic assays were performed at 37 °C. The choice of the buffer used in each case was related to the stability of the substrates and the products, while the concentration of the substrates and the auxiliary enzymes was always high enough to ensure maximum activity of the studied enzymes under the conditions of the assays. In each case, the background activity observed when no substrate is added to the reaction mixture was subtracted from the total activity. Moreover, we checked that no changes in the signal were observed before addition of the cell-free extracts. The details of each enzymatic assay are described in Supplementary Material 5.

Data availability

All the relevant data and scripts are provided as supplementary material.

## Results

### Expression of sucP enabled growth on sucrose, but deletions of *adhE*, *adhP*, *ldhA*, *pta*, and *mhpF* suppressed anaerobic growth

For enabling the sucrose uptake and catabolism in *E. coli* K-12, we chose the episomal expression of the foreign genes *cscB* and *cscK* from *E. coli* W combined with the *cscA* gene from *E. coli* W or the *sucP* gene from *B. adolescentis*. The processes enabled by the proteins encoded by these genes are presented in Fig. 1. Although growth on sucrose had been reported after expressing only the *cscA* gene (Sahin-Toth et al. 1999; Tsunekawa et al. 1992), the observed growth rates were much lower than the obtained expressing a sucrose transporter (Bruschi et al. 2012). Therefore, our rationale to include the expression of the *cscBK* genes next to the expression of *cscA* or *sucP* was to increase the active uptake and quick funneling of the sucrose into fructose-6-phosphate.

The hosting cells were from an *E. coli* K-12 mutant strain with multiple gene deletions (Δ*adhE* Δ*adhP* Δ*ldhA* Δ*pta* Δ*mhpF*). These deletions were previously performed with the aim of minimizing the formation of by-products (acetate, lactate, and ethanol) under oxygen-limited conditions.

Batch cultures at 37 °C, in aerobic or anaerobic conditions, were monitored for up to seven days. The different outcomes are shown in Table 2. In the strains carrying the plasmid pUC19-*cscKB*, no growth on sucrose was observed. This result confirms that, without the introduction of some gene encoding for an enzyme catalyzing the breakdown of the sucrose—either a hydrolase or a phosphorylase—growth of *E. coli* K-12 on sucrose is not possible.

**Table 2:** Growth/no growth using sucrose as the sole carbon source, in aerobiosis and anaerobiosis, for the different recombinant strains obtained combining two different genetic backgrounds with three different plasmids

Strain	Condition	Outcome
TUD37	Aerobic	No growth
TUD66	Aerobic	Growth
TUD69	Aerobic	Growth
TUD37	Anaerobic	No growth
TUD66	Anaerobic	Growth
TUD69	Anaerobic	Growth
TUD38	Aerobic	No growth
SUC-HYD	Aerobic	Growth
SUC-PHOSP	Aerobic	Growth
TUD38	Anaerobic	No growth
SUC-HYD	Anaerobic	No growth
SUC-PHOSP	Anaerobic	No growth

Regarding the growth/no-growth pattern observed under anaerobiosis, it is possible to conclude that the deletions of the genes *adhE*, *adhP*, *ldhA*, *pta*, and *mhpF* were sufficient to abolish growth under the tested conditions. Because we were interested in the study of the sucrose metabolism under oxygen limitation while maintaining low (or zero) by-product formation rates, our chosen approach was the study of the biomass-specific consumption and production rates in strains with the Δ*adhE* Δ*adhP* Δ*ldhA* Δ*pta* Δ*mhpF* deletions using oxygen-limited continuous cultures.

### Construction of an ad hoc in silico metabolic model

For calculation of the biomass-specific conversion rates and possible flux distributions, the biomass composition and the network stoichiometry are very relevant (Dikicioglu et al. 2015). For this reason, the metabolic model constructed by Taymaz-Nikerel and co-workers (Taymaz-Nikerel et al. 2010) was adapted for the design and analysis of the continuous cultures on sucrose (Supplementary Material 2 and the supplementary files *iKOGmaker.m*, *iKOG.mat*, and *Ecolicore.mat*). This model was chosen because (i) it contains experimentally validated estimates for the energetic parameters P/O ratio (*δ*, in mol of ATP/mol of O), growth-dependent ATP expense (*Kx*, in mol of ATP/C-mol_biomass_), and ATP expense for growth-independent maintenance (*m*_*ATP*_, in mol of ATP/C-mol_biomass_/h); and (ii) it contains experimentally determined biomass composition data for the employed dilution rate. However, some modifications were implemented in the original model to represent the genetic modifications present in the constructed strains. For example, the fluxes through the reactions catalyzed by the ethanol dehydrogenase, acetaldehyde dehydrogenase, lactate dehydrogenase, and phosphotransacetylase were set to zero to represent the effect of the gene deletions Δ*adhE*, Δ*adhP*, Δ*mhpF*, Δ*ldhA*, and Δ*pta*. Moreover, the in silico model was further adapted to account for the plasmidial DNA and plasmid-encoded protein burdens in the mutant strains. For these corrections, the DNA and protein sequences, the contribution to the cellular weight of the plasmid-encoded beta-lactamase, and the observed specific sucP activities (12.41 ± 0.45 μmol/min/mg_soluble protein_ in SUC-PHOSP; 0.05 ± 0.04 μmol/min/mg_soluble protein_ in SUC-HYD) were considered.

Flux balance analysis (FBA) using the software Constraints Based Reconstruction and Analysis (COBRA, version 2.0.6) (Schellenberger et al. 2011) for MATLAB (MathWorks, USA) was employed for both the experimental design and the estimation of the metabolic fluxes in the cells grown in the continuous cultures. To predict the metabolic fluxes in the cells growing in the continuous cultures, minimization of the sucrose uptake rate was used as the objective function (see supplementary file *predictionsHYDvsSUCP.m*). To estimate the actual metabolic fluxes, reconciled specific consumption and production rates were employed as constraints, and maximization of the ATP production was used as the objective function. Maximization of ATP production has been reported as a reliable objective function when cells are growing in continuous cultures (Schuetz et al. 2007; van Gulik and Heijnen 1995).

### Sucrose utilization under oxygen-limiting conditions

The experimentally determined specific consumption/production rates with their associated errors (Supplementary Material 6 and supplementary files *qratesHydrolase.m*, *qratesPhosphorylase.m*, *SucHYD.xls*, and *SUCP.xls*) formed the input used to estimate reconciled rates (Table 3; and supplementary files *data_reconciliation_Hydrolase.m*, *data_reconciliation_SucP.m*, and *reconciled rates.xls*), consistent with the carbon and electron conservation principle. Results obtained by other groups while studying *E. coli* MG1655 and *E. coli* mutants with gene deletions similar to the employed in this study, using continuous cultures, are included in Table 3 for comparison.

**Table 3:** Reconciled specific rates during the growth in oxygen-limiting continuous cultures. For comparison, previously published data of different *E. coli*, growing on continuous cultures, are included

	SUC-HYD	SUC-PHOSP	K-12 MG16551	GJT001 (wild type)^2^	YBS125 (Δ*ackA* Δ*pt*	YBS132 (Δ*ackA* Δ*pta* Δ*ldhA*)^2^
Oxygen availability/carbon source(s)	O_2_ limiting/Sucrose	O_2_ limiting/Sucrose	Fully aerobic/Glucose	Anaerobic/LB plus glucose	Anaerobic/LB plus glucose	Anaerobic/LB plus glucose
Specific rates (mmol/g_CDW_/h)						
Glucose	–	–	-0.77	3.55	5.39	7.24
Sucrose	− 0.4157 ± 0.0054	− 0.505 ± 0.0117	–	–	–	–
O_2_	− 2.3177 ± 0.0298	− 3.3428 ± 0.1259	− 1.97*	–	–	–
CO_2_	2.4354 ± 0.0295	3.3907 ± 0.1268	2.19*	n.i.	n.i.	n.i.
Formate	0	0.0348 ± 0.0072	n.d.	5.89	0.56	12.1
Acetate	0.0036 ± 0.0002	0.0066 ± 0.0004	n.d.	3.03	0.15	0.23
Lactate	0.0225 ± 0.0007	0.0129 ± 0.0008	n.d.	0.18	8.29	0.05
Pyruvate	0.0372 ± 0.0076	0.1119 ± 0.0185	n.d.	0	0.25	0.89
Succinate	0.0773 ± 0.0127	0.0831 ± 0.0132	n.d.	0.85	1.16	0.43
Ethanol	n.d.	n.d.	n.d.	2.86	0.41	11.9
Other parameters						
Biomass (h^−1^)	0.0503 ± 0.0005	0.0468 ± 0.0005	0.049*	0.1	0.1	0.1
Yield (g_CDW_/g_substrate_)	0.3535 ± 0.0057	0.2708 ± 0.0069	0.426*	0.156	0.103	0.077
Yield (C-mol_biomass_/C-mol_substrate_)	0.4125 ± 0.0067	0.3188 ± 0.0081	0.522*	0.191	0.13	0.094

*n.d.* not detected, *n.i.* not informed

^1^Data from Taymaz-Nikerel and co-workers (Taymaz-Nikerel et al. 2010). It was considered M_w_^CmolX^ = 24.56 g_CDW_/CmolX

^2^Data from Yang and co-workers (Yang et al. 1999). It was considered M_w_^CmolX^ = 24.56 g_CDW_/CmolX

The carbon and electron recoveries were above 94% (Supplementary Material 7, Supplementary Material 9, Table S3, Table S6, Table S7, Table S8, and Table S9). Considering the extracellular concentrations measured for sucrose, formate, acetate, lactate, pyruvate, and succinate, it was possible to account for 90–95% of the total organic carbon (TOC) present in the filtrate (cell-free) samples obtained from the bioreactors (Supplementary Material 7, Table S4). For this reason, it was assumed that no other metabolites, beyond the measured ones, were produced in meaningful amounts.

Sabri and co-workers observed secretion of fructose into the medium in an *E. coli* W mutant strain where the *cscK* gene was knocked out (Sabri et al. 2013). However, we did not detect glucose or fructose in the samples from extracellular medium (data not shown). The heterologous expression of *cscK* plus the native hexokinase activity probably guaranteed a quick phosphorylation of the fructose resulting from the sucrose hydrolysis/phosphorolysis, preventing its accumulation and leakage to the extracellular medium.

The by-product generation rates represented similar percentages of the consumed carbon (10% and 12%) and electrons (9% and 11%) in SUC-HYD and SUC-PHOSP, respectively. However, some small differences in the by-product profiles were observed between the strains (Table 3).

To assess the potential impact of phosphate on the observed phenotypes, the extracellular concentrations of phosphate were measured. Results indicated similar concentrations in the cultures of SUC-PHOSP (2.6 mM) and SUC-HYD (2.2 mM). These values are far above the critical concentration triggering the *pho* regulon (4 μM, (Lamarche et al. 2008)) and should be saturating for all the known phosphate transporters (Harris et al. 2001; Willsky and Malamy 1980). Therefore, extracellular phosphate concentrations should not have affected the phosphate uptake.

Strikingly, the biomass yield observed for SUC-PHOSP was lower than the observed for SUC-HYD (Table 3 vs Fig. S1). A deeper analysis, considering the effects of the genetic differences between these strains and the different by-product profiles observed during the growth under oxygen limitations, was performed.

With the in silico model corrected to account for the plasmid and recombinant protein burdens (Supplementary Material 10, supplementary file *Biomass composition.xls*), using the reconciled specific consumption/production rates as constraints, and setting the production of ATP as the objective to be maximized, the distributions of the metabolic fluxes were calculated using FBA (see supplementary files *SucHYD_with_burden.m* and *SucPHOSP_with_burden.m*). The upper and lower boundaries for the experimentally determined fluxes were constrained considering the reconciled errors. The calculated metabolic flux distributions are reported in the supplementary file *metabolic fluxes distributions.xls*.

Given the estimated biomass-specific conversion rates, the maximum growth-independent ATP consumption (maintenance) in SUC-HYD was 0.117 mol of ATP/C-mol_biomass_/h, while in SUC-PHOSP it was 0.205 mol of ATP/C-mol_biomass_/h. These values are, respectively, 30% and 130% higher than the upper limit of the growth-independent maintenance ATP expense of 0.075 ± 0.015 mol of ATP/C-mol_biomass_/h estimated by Taymaz-Nikerel and co-workers for the *E. coli* K-12 MG1655 wild type growing on glucose under fully aerobic conditions in a similar growth medium (Taymaz-Nikerel et al. 2010). On the other hand, Gonzalez and co-workers estimated a value of 0.054 mol of ATP/C-mol_biomass_/h for *E. coli* BW25113 (apparently wrongly typed as BW21135) anaerobically growing on glucose (Gonzalez et al. 2017).

Two important conclusions can be obtained from these calculations: (i) the growth of *E. coli* K-12–derived strains on sucrose under oxygen-limiting conditions, despite the kind of enzymatic mechanism for the breakdown of the disaccharide, implied a large increase in the ATP expenses generically named “maintenance”; and (ii) because biomass compositions, plasmid and protein burdens, P/O ratios, and growth-dependent ATP expenses in SUC-HYD and SUC-PHOSP are similar (Supplementary Material 8, Fig. S3, Fig. S4, Table S5), with the available data it is not possible to unmistakably identify the biological process(es) responsible for the difference between the biomass yields observed in such strains. However, further analyses pointed to an increase in ATP futile cycles triggered by the use of sucrose under oxygen-limiting conditions, being these futile cycles higher in SUC-PHOSP.

### Evidence pointing to ATP futile cycles of different extensions in SUC-HYD and SUC-PHOSP

One of the best-known futile ATP cycles is caused by the simultaneous operation of the reactions catalyzed by the fructose-1,6-bisphosphatase (FBPase) and the phosphofructokinase (PFK). The specific FBPase activities in samples of SUC-HYD and SUC-PHOSP, obtained during the continuous cultures, were measured. The activities from aerobic batch cultures of *E. coli* K-12 MG1655 grown on 5 g/L of acetate and 5 g/L of glucose were included for comparison. The results showed that the activity of FBPase was approximately eight times higher in SUC-PHOSP than in SUC-HYD (Table 4). Remarkably, the specific FBPase activities in SUC-HYD and SUC-PHOSP were 10–70 times higher than those observed in *E. coli* K-12 MG1655 grown in gluconeogenic conditions, i.e., a situation in which FBPase is required to survive.

**Table 4:** Specific fructose-1,6-biphosphatase activity

Strain	Growth conditions	Carbon source	Specific activity (U/mg _soluble protein_)
*E. coli* K-12 MG1655	Aerobic shake flask	Glucose	0.44 ± 0.08
*E. coli* K-12 MG1655	Aerobic shake flask	Acetate	0.98 ± 0.05
SUC-HYD	chemostat	Sucrose	9.41 ± 1.47
SUC-PHOSP	chemostat	Sucrose	72.32 ± 4.27

1 U = μmol_NADPH_/min

**Fig. 2:**
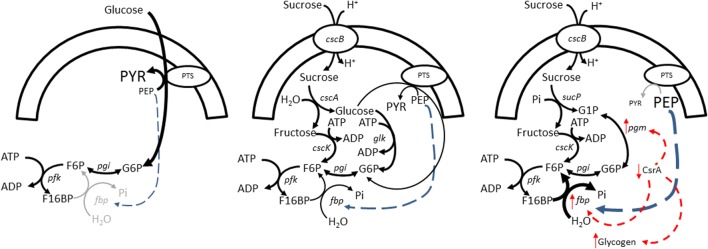
Possible regulatory mechanisms leading to an increment in the flux through the futile cycle PFK/fructose-1,6-biphosphatase. The names of the intracellular enzymes and the sucrose/H^+^ symporter are represented with the names of their encoding genes. Left, uptake of glucose through the PTS. The concentration of PEP is small and fructose-1,6-biphosphatase is barely active. Middle, in the strain SUC-HYD growing on sucrose, a fraction of the internally released glucose is phosphorylated by the PTS. However, the concentration of PEP is higher than that in cells growing on glucose and consequently the fructose-1,6-biphosphatase activity is higher. Right, in the case of the strain SUC-PHOSP, the activity of PTS should be absent and the concentration of PEP should be even higher leading to a stronger fructose-1,6-biphosphatase activity. Still, it is likely that the activity of the CsrA protein be smaller in SUC-PHOSP leading to a higher PGM activity, a higher fructose-1,6-biphosphatase activity, and a larger accumulation of glycogen. PTS, phosphotransferase system; G6P, glucose-6-phosphate; F6P, fructose-6-phosphate; G1P, glucose-1-phosphate; F16BP, fructose-1,6-biphosphate; Pi, inorganic phosphate

In any case, the differences in FBPase activities between SUC-HYD and SUC-PHOSP detected in the enzymatic assay reflect differences in the transcription and/or the post-translational level rather than differences caused by allosteric effects. Therefore, we should expect some difference in the activities of the proteins regulating the sugar metabolism. Following this clue, it has been shown that deletion of the *csrA* gene (encoding for the carbon storage regulator A, CsrA) leads to overexpression of phosphoglucomutase (PGM) (Sabnis et al. 1995). Because PGM is required in SUC-PHOSP for the conversion of glucose 1-phosphate into glucose 6-phosphate (G6P), if CsrA activity in SUC-PHOSP were lower, the consequent increment in PGM activity could be beneficial for the rate of sucrose assimilation in SUC-PHOSP. However, the decrease in CsrA activity also led to an increase in the expression of FBPase and PEP synthase (Sabnis et al. 1995), potentially leading to ATP futile cycles (Fig. 2).

To verify this prediction, the PGM activity was measured in samples of SUC-HYD and SUC-PHOSP taken from the oxygen-limited cultures. For comparison, the PGM activity in cells from the strain TUD69 aerobically grown on sucrose (PGM is required to survive in these conditions) was also measured. The results showed that the PGM activity was much higher in the sample taken from SUC-PHOSP compared with that from SUC-HYD and TUD69 (Table 5). This observation is consistent with the hypothesis of lower CsrA activity in the cells of SUC-PHOSP, provoking higher expression of FBPase and PEP synthase, leading to higher ATP futile cycles.

**Table 5:** Specific phosphoglucomutase activity

Strain	Growth conditions	Carbon source	Specific activity (U/mg _soluble protein_)
TUD69	Aerobic shake flask	Sucrose	0.074 ± 0.012
SUC-HYD	chemostat	Sucrose	0.013 ± 0.002
SUC-PHOSP	chemostat	Sucrose	9.872 ± 0.870

1 U = μmol_NADPH_/min

Finally, the decrease in CsrA activity should also lead to a larger accumulation of glycogen. To test this second prediction, the concentrations of glycogen in SUC-HYD and SUC-PHOSP were measured. Glycogen content corresponded to 0.8% of the biomass in SUC-PHOSP and 0.1% in the case of SUC-HYD. The reported glycogen content of *E. coli* MG1655 growing on glucose in an aerobic carbon-limited chemostat at a dilution rate of 0.05 h^−1^ is about 0.8% (Taymaz-Nikerel et al. 2010), a value in agreement with our result for SUC-PHOSP. FBA was employed to evaluate the impact on the ATP consumption of the observed difference in glycogen content. As a result, the increase in ATP consumption to account for the observed difference in glycogen content was only 0.08 mmol_ATP_/g_CDW_/h. Therefore, the difference in glycogen content does not explain the differences in biomass yields between SUC-HYD and SUC-PHOSP. Nevertheless, the higher glycogen content in SUC-PHOSP is consistent with the hypothesis of lower CsrA activity in that strain.

## Discussion

There is a long background information regarding the functional characterization of the genes enabling sucrose uptake and hydrolysis from different species (Alaeddinoglu and Charles 1979; Bockmann et al. 1992; Jahreis et al. 2002; Sabri et al. 2013). Bruschi and co-workers summarized the efforts focused in the expression of such genes in *E. coli* strains incapable to grow on sucrose (Bruschi et al. 2012). However, to the best of our knowledge, this the first time that the energetic impact of sucrose phosphorolysis in *E. coli* was quantified.

Kim and co-workers reported the growth of *E. coli* JM-109 in M9 plates supplemented with raffinose, melibiose, or sucrose (at 40 g/L) after the introduction of a plasmid carrying the putative *sucP* gene from *Bifidobacterium longum* (Kim et al. 2003). The encoded protein was semi-purified and showed remarkable similarities with other sucrose phosphorylases. Nevertheless, these authors only reported a qualitative result (growth on plates where sucrose was the sole carbon source) without reporting any growth rate. Moreover, the enzymatic assays to verify sucrose phosphorolysis were performed with synthetic analogs instead of sucrose.

In another of the few reported cases of *E. coli* growth driven by sucrose phosphorolysis, De Bruyn and co-workers replaced the chromosomal *cscA* gene in *E. coli* W by the *sucP* gene from *Bifidobacterium adolescentis* to enhance the production of UDP-glucose (derived from glucose-1-phosphate coming from sucrose phosphorolysis) (De Bruyn et al. 2015). The production of glycosylated phenolic acids derived from the UDP-glucose and externally supplied phenolic compounds indicated that sucrose phosphorolysis indeed happened.

According to Bruschi and co-workers (Bruschi et al. 2012), it is enough to express the *cscBKA* genes from *E. coli* W to observe the growth on sucrose in *E. coli* K-12 MG1655. Therefore, we considered not necessary to express the *scrY* gene from the plasmid pUR400, encoding for a sucrose porin anchored in the outer membrane (Schmid et al. 1988).

Although anaerobic growth of *E. coli* K-12 is theoretically possible without producing acetate, ethanol and lactate as long as hydrogen, acetaldehyde, and succinate are produced, the experimental results indicated that the deletions of the genes *adhE*, *adhP*, *ldhA*, and *mhpF* severely limited the capacity to re-oxidize NADH, provoking a redox imbalance. Moreover, it is possible that a reduced flux from acetyl-CoA to acetate, caused by the *pta* deletion, affected the substrate-level production of ATP in the reaction catalyzed by the acetate kinase. Still, it is possible that the use of sucrose triggered ATP-dissipation mechanisms impeding the biomass formation under anaerobic conditions.

While the redox imbalance provoked by such genetic deletions could be solved with the generation of (commercially interesting) reduced products such as ethanol, lactic acid, or polyhydroxyalkanoates, the decrease in the substrate-level production of ATP and by-product suppression is a frequent problem in anaerobic bioprocesses. Because the deletions of the *adhE*, *adhP*, *ldhA*, *pta*, and *mhpF* genes impeded the growth under fully anaerobic conditions, the study of the energetic impact of using sucrose was performed under continuous oxygen-limiting conditions.

The observed biomass yields were lower than expected, even considering the cost of sucrose:H^+^ symport and the plasmid and protein burdens. Remarkably, the biomass yield of the strain SUC-PHOSP was unexpectedly smaller than that observed for the strain SUC-HYD, with a relatively larger fraction of the sucrose being fully catabolized to CO_2_.

Evidence was found consistent with the idea of ATP futile cycles as the reason behind the difference between the expected and the observed biomass yields. Although the FBPase activity in SUC-PHOSP was near eight times higher than that in SUC-HYD, it is known that the *in vivo* FBPase activity is highly regulated by allosterism (Hines et al. 2006). Therefore, with the available data, it is impossible to state that the *in vivo* metabolic flux through FBPase was indeed higher in SUC-PHOSP. Nevertheless, we can speculate about the contributions of (i) the gene deletions, (ii) the oxygen limitation, (iii) the carbon source, (iv) the intracellular phosphate concentrations, and (v) the mechanism to break down the sucrose.

The deletion of genes encoding for enzymes involved in the by-product generation is a common practice in biotechnology (Förster and Gescher 2014; Ruiz et al. 2012). However, it has been observed a decrease in the biomass yield after the deletion of *pflB* (Wang et al. 2010), *pflA* (Matsuoka and Shimizu 2014), *pta* (Matsuoka and Shimizu 2014), *adhE* (Matsuoka and Shimizu 2014), and *ldhA* (Yang et al. 1999). On the other hand, De Mey and co-workers did not find differences in the maintenance ATP (around 0.088 mol of ATP/C-mol_biomass_/h) between *E. coli* MG1655 and its mutant lacking the genes *ackA*, *pta*, and *poxB* while growing anaerobically on glucose as the sole carbon source (De Mey et al. 2007).

Regarding the influence of the oxygen limitation (ii), it has been reported that the specific activities of both FBPase and PFK increase in comparison with fully aerobic conditions (Peng and Shimizu 2003). Therefore, the oxygen limitation could itself have an effect on the increase in FBPase activity. This explanation is consistent with the higher FBPase activities observed in the samples coming from the oxygen-limited cultures by comparison with the level in *E. coli* K-12 MG1655 growing in shaking flasks.

Regarding the carbon source employed (iii), an increased PEP to pyruvate ratio has previously been observed in *E. coli* MG1655 growing on sucrose (Bettenbrock et al. 2007), and it has been reported that FBPase is allosterically activated by PEP (Hines et al. 2006). Therefore, the growth on sucrose could trigger an increment in the concentration of PEP leading to an increment in the FBPase activity. Because the expression of FBPase is itself affected by the oxygen limitation, the combination of the use of sucrose with oxygen limitation could result in even larger FBPase activities compared with the levels registered growing aerobically on glucose or acetate, explaining the lower biomass yields observed in both SUC-HYD and SUC-PHOSP by comparison with the expected values.

None of the previous discussed factors, however, explains the unexpected lower biomass yield observed in SUC-PHOSP by comparison with SUC-HYD. One of the factors that could be indeed different between these strains, leading to different network operations, is the intracellular free phosphate concentrations (iv). Phosphate is a substrate for the reaction catalyzed by the SucP and, at the same time, it is one of the products of the reaction catalyzed by the FBPase. Thus, it is possible that in those cells with higher FBPase activity, the released phosphate had a positive impact on the rate of sucrose phosphorolysis, probably easing a metabolic bottleneck. Another source of phosphate could be the flow through the methylglyoxal pathway. In this pathway, one dihydroxyacetone-phosphate is converted into pyruvate and inorganic phosphate is realized, while no ATP is produced. In that case, although more phosphate is released, the ATP yield is affected. Moreover, methylglyoxal toxicity could have additional negative effects.

Another process affected by the cytoplasmic phosphate concentration is the relative use of water or phosphate as nucleophile in the sucrose breakdown catalyzed by the SucP. However, if water fully replaced phosphate during the sucrose breakdown in SUC-PHOSP, we should have seen a biomass yield similar to SUC-HYD, but it was not the case. Still, it is possible that different intracellular concentrations of free phosphate in these two strains provoked different H^+^:ATP stoichiometries in the proton pumps (D’Alessandro et al. 2008). Nevertheless, given the multiple binding interactions between phosphate ions and intracellular molecules, the accurate measuring of cytoplasmic free phosphate concentrations is extremely challenging and requires significant additional effort beyond the scope of this research.

Next to the potential differences in the intracellular free phosphate concentrations, the differences between the mechanisms to break down the sucrose in SUC-HYD and SUC-PHOSP (v) could result in different metabolomics profile. Because PTS has the capacity to phosphorylate both extracellular and intracellular glucose (Postma et al. 1993), the PTS activity was probably higher in SUC-HYD than in SUC-PHOSP, leading to lower concentration of PEP in SUC-HYD and, consequently, a lower FBPase activity by comparison with SUC-PHOSP (Fig. 2). A report showing that deletion of the *ptsI-ptsH-crr* genes in *E. coli* increased the concentration of PEP (Flores and Xiao 1996) is consistent with this explanation.

It was finally found evidence pointing to different levels of CsrA activities in SUC-HYD and SUC-PHOSP. Although a decrease in CsrA activity could improve PGM activity, making easier the assimilation of carbon in SUC-PHOSP, it could also increase the activities of futile ATP cycle–related enzymes such as FBPase and PEP synthase.

Overall, our results confirm that the implementation of sucrose consumption and reduction of by-product in *E. coli* K-12 is technically easy. However, they also showed an unexpected increase in ATP expenses under such conditions, being larger in the strain expressing a sucrose phosphorylase. The found evidence points to the operation of an ATP futile cycle due to the simultaneous operation of the phosphofructokinase(s) and the FBPase. One theoretically possible way to eliminate this futile cycle is the deletion of the genes *pfkA* and *pfkB*, enforcing the glycolytic flow through reactions belonging to the Entner-Doudoroff pathway or the pentose-phosphate pathway (Hollinshead et al. 2016). However, this approach should reduce the ATP yield. In that case, the only way to increase the ATP yield is with the further implementation of some heterologous glycolytic pathway, such as the bifid shunt. On the other hand, the deletion of the genes *fbp* and *glpX*, encoding for fructose-1,6-biphosphatases, seems to be an easier solution but the resulting organisms will be unable to survive in gluconeogenic conditions. Therefore, the best way to suppress this potential ATP futile cycle will depend on the specific application.

Anyways, it is extremely complicated to eliminate all the possibilities for futile cycles and probably is not recommendable either. Next to the case discussed above, futile cycles can be established by the simultaneous activity of phosphoenolpyruvate synthase (EC 2.7.9.2) and pyruvate kinase (EC 2.7.1.40) (Patnaik et al. 1992) or phosphoenolpyruvate carboxykinase (EC 4.1.1.49) and phosphoenolpyruvate carboxylase (EC 4.1.1.31) (Chao and Liao 1994) among others. ATP dissipation seems to be present in many organisms, contributing to the adjustment between the rates of catabolic supply and anabolic demand (Russell 2007) and could be also important as a defense mechanism against oxidative stress (Adolfsen and Brynildsen 2015).

With the obtained data, it is not possible to distinguish the individual contributions of the by-product suppression, the oxygen limitation, and the use of sucrose to the observed increment in the ATP expense. Therefore, further studies focused in the observation of the individual effects of these factors are required.

It is surprising that, despite the frequent observation of decrease in the biomass yields after the deletions of genes involved in by-product generation, the mechanism(s) explaining the fall in the biomass yields are seldom discussed. Wang and co-workers proposed that ATP dissipation was probably a response to a relative excess of ATP respect to the amino acids and other building blocks in the mutant lacking *pflB* (Wang et al. 2010). It is possible that those gene deletions provoked metabolic bottlenecks, triggering the expression of genes involved in futile cycles. As a matter of fact, in the cases of the deletion of *pflA*, *pta*, and *adhE*, increments in the concentration of several central pathway metabolites were observed, including fructose-1,6-bisphosphate (Matsuoka and Shimizu 2014), which is widely acknowledged as a signaling metabolite (Kochanowski et al. 2013).

Regarding the potential effect of oxygen limitation in the observed decrease of the ATP yields, it seems interesting to study the impact on the ATP yields of the introduction of production pathways capable to re-oxidize the NADH under anaerobic conditions. One possibility could be the introduction of a NADH-consuming polyhydroxybutyrate pathway (de Las Heras et al. 2016; Ling et al. 2018).

Finally, the metabolic impact of the use of sucrose in *E. coli* MG1655 derivatives requires attention. While studying the sucrose metabolism of an *E. coli* strain naturally capable to grow on sucrose, Jahreis and co-workers found evidence indicating an evolutionary recent acquisition of the genes enabling the use of this disaccharide (Jahreis et al. 2002). The catabolism of sucrose using a hydrolase or a phosphorylase implies intracellular concentrations of fructose and glucose (or glucose-1-phosphate) larger than the expected for the catabolism of glucose. It is possible that an increment in the concentration of sugar phosphates induces the (energetically less efficient) methylglyoxal pathway. To avoid this situation, an increment in the phosphoglucomutase activity could accelerate the funneling of carbon into the Embden-Meyerhof pathway, preventing the accumulation of sugar phosphates. Thus, further studies focused on the changes in the metabolome when the carbon source shifts from glucose to sucrose should help us to understand the mechanisms behind the unexpected decrease in biomass yield after the implementation of sucrose consumption in *E. coli* K-12.

## Electronic supplementary material

ESM 1 (DOCX 436 kb)
