# Supplementary material for: Metabolism of sucrose in a non-fermentative Escherichia coli under oxygen limitation
Source: Appl Microbiol Biotechnol. 2019 May 31;103(15):6245–56. doi: 10.1007/s00253-019-09909-6 (PMC6616217; doi:10.1007/s00253-019-09909-6)
Supplement: Supplementary file 1 — (DOCX 436 kb) [file 253_2019_9909_MOESM1_ESM.docx]

Applied Microbiology and Biotechnology

Metabolism of sucrose in a non-fermentative *Escherichia coli* under oxygen limitation

Authors: Karel Olavarria, Albert Fina, Mariana I. Velasco, Mark C. M. van Loosdrecht and Sebastian Aljoscha Wahl

corresponding authors:

[K.OlavarriaGamez@tudelft.nl](mailto:K.OlavarriaGamez@tudelft.nl) (ORCID 0000-0003-0435-7640)

[S.A.Wahl@tudelft.nl](mailto:S.A.Wahl@tudelft.nl) (ORCID 0000-0003-2120-1859)

**Main Supplementary Text**

**The reproducibility of the here-presented analyses requires the use of different material provided as supplementary files.**

Besides this **“Main Supplementary Text”** (which contains the Supplementary materials mentioned in the main texts, data, Supplementary Tables and Supplementary Figures), we provide further supplementary files:

1. Three files contain the DNA sequences of three constructed plasmids: **pUC19-cscABK**, **pUC19-cscBK** and **pUC19-cscBK-sucP**. These three files are in GeneBank format.
2. The file named **iKOGmaker** is a MATLAB script to generate the *in-silico* model **iKOG.mat**. This script calls the *in-silico* model **Ecolicore.mat**. To run this script the software COBRA Toolbox for MATLAB is required. The Ecolicore.mat was downloaded from the BiGG database (UCSD) and it is also provided.
3. The Excel file named **Biomass composition** contains the calculations required to assess the composition of the lean and the plasmid+protein burdened biomass.
4. The MATLAB file named **predictionsHYDvsSUCP** contains the code to make the prediction of the by-product generation profile and the expected specific sucrose consumption rates for the strains SUC-HYD and SUC-PHOSP. This prediction requires the use of the software COBRA Toolbox for MATLAB.
5. The Excel file named **calculating required stirring** contains the calculations of the required stirring rates to achieve the required oxygen transference rates.
6. The Excel files named **SUCHYD** and **SUCP** contain the raw data acquired during the continuous cultivations of SUC-HYD and SUC-PHOSP under oxygen limiting conditions. The MATLAB files named **qratesHydrolase** and **qratesPhosphorylase** read these raw data and calculate the non-reconciled biomass specific conversion rates.
7. The MATLAB files named **data_reconciliation_Hydrolase** and **data_reconciliation_SucP** calculate the reconciled biomass specific conversion rates using as input the non-reconciled biomass specific conversion rates with their associated experimental errors, and the biomass composition calculated considering the DNA plus Protein burdens. The Excel file named **reconciled rates** contains calculations verifying the consistency of the reconciled biomass specific conversion rates.
8. The MATLAB files named **SucHYD_with_burden** and **SucPHOSP_with_burden** contain the scripts to calculate the metabolic fluxes distributions in the strains SUC-HYD and SUC-PHOSP, using as input the reconciled biomass specific conversion rates. The fluxes were calculated using Flux Balance Analysis. To calculate the metabolic fluxes the software COBRA Toolbox for MATLAB is required. The Excel file named **metabolic fluxes distributions** contains the result of the Flux Balance Analysis.

Index

[Supplementary material 1: Construction of the plasmids pUC19-csc*AKB*, pUC19-*cscKB* and pUC19-*cscKB*-*sucP* 3](#_Toc6311282)

[Supplementary material 2: Adapting the *in-silico* model to represent the strains SUC-HYD and SUC-PHOSP 5](#_Toc6311283)

[Supplementary material 3: Experimental design for the oxygen-limiting continuous cultures 7](#_Toc6311284)

[Supplementary material 4: Setup and operation of the continuous cultures 12](#_Toc6311285)

[Supplementary material 5: Analytical methods 13](#_Toc6311286)

[Supplementary material 6: Non-reconciled rates observed in the continuous cultures of SUC-HYD and SUC-PHOSP under oxygen-limiting conditions. 16](#_Toc6311287)

[Supplementary material 7. TOC/TN measurements. Carbon balance of the by-products, C fraction in biomass and N:C ratio in biomass 17](#_Toc6311288)

[Supplementary material 8. Codon usage analysis 19](#_Toc6311289)

[Supplementary material 9. Overall carbon and electron balances 21](#_Toc6311290)

[Supplementary material 10: Modifications in the *in-silico* model to consider plasmid and protein burden 25](#_Toc6311291)

Supplementary material 1: Construction of the plasmids pUC19-csc*AKB*, pUC19-*cscKB* and pUC19-*cscKB*-*sucP*

Routine molecular biology procedures were performed according with standards protocols (Sambrook et al. 1983). Genetic backgrounds of the strains are summarized in Table 1 (main document) and primers employed are listed in Table S 1.

The *cscAKB* genes from *E. coli* W (strain DSM-20083 (ATCC 15703), DSMZ, Germany), encoding for a sucrose hydrolase, a fructose kinase and a sucrose/H+ symporter respectively were amplified using genomic DNA as template, using the primers cscAKB_Xba_FW and cscAKB_Bam_RV, and the Phusion High-Fidelity DNA Polymerase (New England Biolabs, U.S.A.). The resulting amplicon was cleaned using the GeneJET Purification Kit (Thermo Fisher Scientific, USA) and cut using the restriction enzymes *Bam*HI and *Xba*I (New England Biolabs, U.S.A.). The plasmid pUC19 (Invitrogen, U.S.A.) was restricted in parallel with the same enzymes and the cut vector and PCR product were ligated using DNA T4-ligase (New England Biolabs, U.S.A.). The ligation mixture was employed to transform chemically competent *E. coli* Top10 cells (Invitrogen, U.S.A.) and, after one hour of recovery in lysogenic broth (LB) at 37 °C, the cells were transferred to LB plates supplemented with 0.1 mg/mL of ampicillin. The presence of plasmids carrying the *cscABK* genes was checked by colony-PCR.

The construction of an artificial operon combining the *cscKB* genes from *E. coli* W and *sucP* (encoding for a sucrose phosphorylase) from *Bifidobacterium adolescentis* was performed in two steps. For the first step, the primers csc_Xba_FW and cscKB_Bam_RV were used to amplify the *cscKB* genes of the *csc* operon from *E. coli* W. Afterwards, using a process similar to the employed to obtain the plasmid pUC19-*cscAKB*, the plasmid pUC19-*cscKB* was obtained. For the second step, the *sucP* gene was amplified using genomic DNA from *B. adolescentis* (strain DSM-20083, DSMZ, Germany, ATCC 15703, CCUG 18363, NCTC 11814), the primers sucP_Bam_FW and sucP_Kpn_RV and Phusion High-Fidelity DNA Polymerase. The resulting amplicon and the plasmid pUC19-*cscKB* were restricted with *Bam*HI and *Kpn*I (New England Biolabs, U.S.A.) and the resulting products were cleaned and ligated using the same procedure described above. Some colonies were screened by PCR to confirm the presence of the genes *cscKB*-*sucP*. The obtained plasmids (pUC19-*cscAKB* and pUC19-*cscKB-sucP*) were purified using the QIAprep Spin Miniprep Kit (Qiagen, Germany). The fragments containing the *cscAKB* and *cscKB*-*sucP* genes were sequenced (BaseClear, The Netherlands) to rule-out mutations arising during the molecular manipulations. The (GeneBank-formated) DNA sequences of the plasmids pUC19-csc*AKB*, pUC19-*cscKB* and pUC19-*cscKB*-*sucP* are provided as supplementary files.

Table S 1: Names and sequences of the employed primers. The underlined sequences represent the target for the indicated restriction enzymes.

| Primer | Restriction enzyme | Restriction target underlined |
| --- | --- | --- |
| csc_Xba _FW | *Xba*I | 5’-CATTGCTCTAGAGCCTATATTGCTGAAGGTACAG-3’ |
| csc_Bam_RV | *Bam*HI | 5’-ATTCGCGGATCCGCGTTAACCCAGTAGCCAGAGTG-3’ |
| cscKB_Bam_RV | *Bam*HI | 5’-ATTCGCGGATCCCATCCTGGTTAAGCAAATTTG-3’ |
| sucP_Bam_FW | *Bam*HI | 5’-TAACGCGGATCCATGAAAAACAAGGTGCAGCTC-3’ |
| sucP_Kpn_RV | *Kpn*I | 5’-TTACGGGGTACCTCAGGCGACGACAGGCGGATTG-3’ |

Supplementary material 2: Adapting the *in-silico* model to represent the strains SUC-HYD and SUC-PHOSP

This supplementary material explain the steps to adapt the *in-silico* model published by Taymaz-Nikerel and co-workers (Taymaz-Nikerel et al. 2010) in an *in-silico* model suitable for FBA using the software COBRA (Schellenberger et al. 2011). The script to make this conversion possible (iKOGmaker_with_comments), as well as the resulting *in-silico* model (*iKOG*) can be found as supplementary files.

- The *Ecolicore* model (Orth et al. 2010) was employed as backbone. This model can be freely downloaded from the BiGG database (<http://bigg.ucsd.edu/)> and it is here provided as a supplementary file.
- All but exchange reactions were removed from the *Ecolicore* model.
- Additional exchange reactions were introduced for the following metabolites/molecules: SO_4_^-2^ (sulphur source), sucrose, H_2_ and Biomass.
- The 284 reactions from the model of Taymaz-Nikerel and co-workers were manually introduced. Some of these reactions were modified:

1. To comply with the thermodynamic feasibility, the reaction originally labelled as r44 was re-written as:

- dhap[c] + H^+^[c] + nadph[c] -> glyc3p[c] + nadp[c]

1. According with the experimental results of Sauer and co-workers (Sauer et al. 2004), the mutant Δ*pgi* Δ*udhA* cannot grow on glucose as the sole carbon source because it cannot balance the production and consumption of NADPH. If the membrane-bound transhydrogenase could operate *in vivo* pumping out protons while transferring reducing equivalents from NADPH to NAD, the growth of such mutant on glucose should be feasible. Because the experimental results indicate that the membrane-bound transhydrogenase is not reversible *in vivo*, two separate irreversible reactions were placed instead of the original reversible reaction r284:

- 2 H^+^[e] + nadp[c] + nadh[c] -> 2 H^+^[c] + nad[c] + nadph[c]
- nadph[c] + nad[c] -> nadh[c] + nadp[c]

1. The following reaction was introduced to represent the Entner-Doudoroff pathway:

- 6pgc[c] + h2o[c] -> pyr[c] + g3p[c]

1. Other reactions were added to represent fluxes operating under anaerobic conditions:

- fum[c] + q8h2[c] -> succ[c] + q8[c]
- pyr[c] + coa[c] -> accoa[c] + for[c]
- for[c] + H^+^[c] -> co2[c] + H_2_[c]

1. When necessary, more reactions were added to enable the uptake and assimilation of the sucrose:

- Sucrose/H^+^ symport: H^+^[e] + sucrose[e] <=> H^+^[c] + sucrose[c] (sucrose/ H^+^ symport)
- pi[c] + sucrose[c] <=> g1p[c] + fru[c] (sucrose phosphorylase)
- h2o[c] + sucrose[c] <=> fru[c] + glc-D[c] (sucrose hydrolase)
- atp[c] + fru[c] <=> f6p[c] + adp[c] (fructose kinase)
- atp[c] + glc-D[c] <=> g6p[c] + adp[c] (glucose kinase)

Supplementary material 3: Experimental design for the oxygen-limiting continuous cultures

Beyond the genotype, the choice of suitable experimental conditions is critical to assess the differences between the strains breaking-down the sucrose using a hydrolase or a phosphorylase. These differences should become evident through the comparison between biomass-specific consumption and production rates. Therefore, discriminating measurements required a reliable measure of the biomass concentration. To accomplish this goal, calculations were done to expect biomass concentrations around 5 gCDW/L during the continuous cultures (see below).

Simulations using FBA were performed to estimate the experimental conditions that enhance the expected differences in biomass yield between SUC-HYD and SUC-PHOSP. The oxygen availability (defined as maximum oxygen consumption rate allowed in each simulation) was chosen as the independent variable, while the specific growth rate was fixed to 0.05 h^-1^. The by-products generation rates and the minimal sucrose consumption rate were predicted as a function of the oxygen uptake rate for the strains SUC-HYD and SUC-PHOSP (see **Figure S 1**). As expected, for all the explored levels of oxygen availability, the simulations showed that SUC-PHOSP needed less sucrose than SUC-HYD to grow at the same dilution rate (see **Figure S 1**). The difference in the biomass yield between both strains should increase with decreasing oxygen uptake, which is explained by the increasing fraction of ATP originated from substrate level phosphorylation.

The analysis predicted that, at an oxygen uptake rate of 2 mmol_O2_/g_CDW_/h, only a small amount of pyruvate should be produced while the sucrose consumption rates should be qS_SUC-PHOSP_=0.39 mmol_sucrose_/g_CDW_/h and qS_SUC-HYD_=0.46 mmol_sucrose_/g_CDW_/h (see **Figure S 1**). Because the *pKa* of pyruvate is 2.5, at pH 7.0 the predominat form will be the pyruvate anion, diminishing the possibilities of ATP dissipation due to weak acid cycling. Therefore, if the predictions of the model were fulfilled, with an oxygen uptake rate around 2 mmol_O2_/g_CDW_/h there should be: (1) very small (not) accumulation of by-products and (2) an easily measureable difference (around 20%) in sucrose consumption rates. For experimental simplicity, the sucrose concentration in the feeding solution was equal in both experiments and the expected biomass concentration and required oxygen transfer rate were derived from the calculated yield (FBA) and assumption of full consumption of the substrate.

**
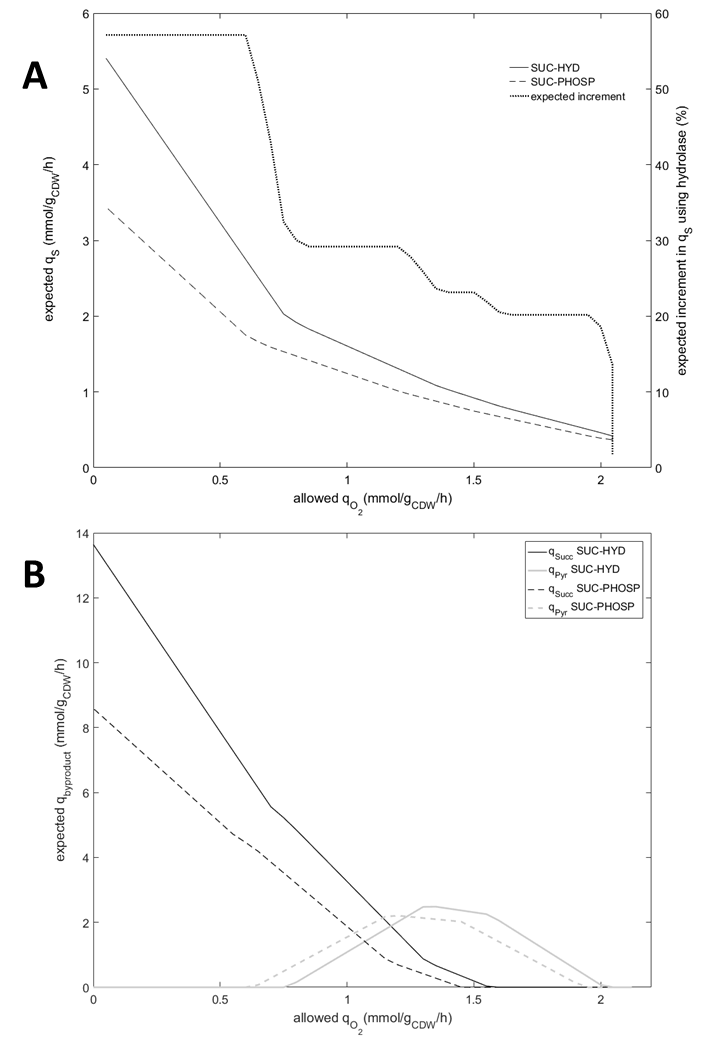
**

Figure S 1: Expect specific sucrose consumption rates (A) and by-product generation rates (B) as a function of the allowed specific oxygen uptake rate for the strains SUC-HYD and SUC-PHOSP. Abbreviations: Succ, succinate; Pyr, pyruvate. The growth dependent ATP expense and the ATP expense for growth-independent maintenance were set to Kx = 0.73 mol of ATP/C-molbiomass, and mATP = 0.09 mol of ATP/C-molbiomass/h, respectively, according with the upper bounds determined by Taymaz-Nikerel and co-workers (Taymaz-Nikerel et al. 2010) for such parameters.

*Determination of the kLa and the bioreactor settings*

According with the *a priori* FBA simulations, for an oxygen uptake rate of 2 mmol_O2_/g_CDW_/h the biomass yields of SUC-HYD and SUC-PHOSP would be 0.3978 g_CDW_/g_sucrose_ and 0.4091 g_CDW_/g_sucrose_ respectively. Using a feeding medium containing 15 g/L of sucrose, and assuming all the fed sucrose will be consumed by the biomass, the biomass concentration should be around 5.88 g/L for SUC-HYD and 6.11 g/L for SUC-PHOSP.

In a chemostat culture under steady state, the oxygen transfer rate (OTR) is equal to the oxygen uptake rate (OUR).

$\boldsymbol{OTR}\boldsymbol{=}\boldsymbol{k}_{\boldsymbol{L}}\boldsymbol{a}\boldsymbol{\cdot}\left( \boldsymbol{C}_{\boldsymbol{O}_{\boldsymbol{L}}}^{\boldsymbol{*}}\boldsymbol{-}\boldsymbol{C}_{\boldsymbol{O}_{\boldsymbol{L}}} \right)\boldsymbol{\cdot}\boldsymbol{V}$ Equation 1

$\boldsymbol{OUR}\boldsymbol{=}\boldsymbol{q}\boldsymbol{O}_{\boldsymbol{2}}\boldsymbol{\cdot}\boldsymbol{C}_{\boldsymbol{x}}\boldsymbol{\cdot}\boldsymbol{V}$ Equation 2

The $k_{L}a$ is the oxygen transfer coefficient (h^-1^), $C_{O_{L}}^{*}$is the concentration of oxygen under saturation conditions (mmol_O2_/L), $C_{O_{L}}$is the actual oxygen concentration in the liquid (mmol_O2_/L); qO_2_ is the oxygen uptake rate (mmol_O2_/g_CDW_/h), C_x_ is the biomass concentration (g_CDW_/L) and V is the volume of the reactor (L).

The $C_{O_{L}}$is almost zero in an oxygen-limited chemostat. Therefore, when the OTR is equal to the OUR, the following formula for the determination of the $k_{L}a$ is derived:

$\boldsymbol{k}_{\boldsymbol{L}}\boldsymbol{a}\boldsymbol{=}\frac{{\boldsymbol{q}\boldsymbol{O}_{\boldsymbol{2}}\boldsymbol{\cdot}\boldsymbol{C}}_{\boldsymbol{x}}}{\boldsymbol{C}_{\boldsymbol{O}_{\boldsymbol{L}}}^{\boldsymbol{*}}}$ Equation 3

where $C_{O_{L}}^{*}$ is the saturation concentration of oxygen in the liquid phase.

The $C_{O_{L}}^{*}$ was calculated using the composition of the air at the inflow and the Henry coefficient (*H_e_*).

$\boldsymbol{H}\boldsymbol{e}_{\boldsymbol{O}_{\boldsymbol{2}}}\boldsymbol{=}\frac{\boldsymbol{C}_{\boldsymbol{O}_{\boldsymbol{L}}}^{\boldsymbol{*}}}{\boldsymbol{P}_{\boldsymbol{O}_{\boldsymbol{2}}}}$ Equation 4

Where $He_{O_{2}}$ is the Henry coefficient at the desired conditions (in mol/m^3^/Pa), $C_{O_{L}}^{*}$ is the saturation concentration of oxygen in the liquid phase (in mol/m^3^), $P_{O_{2}}$ is the partial pressure of oxygen in the gas phase (in Pa). However, the Henry coefficient needed to be corrected for the actual conditions of the assay using the Van’t Hoof’s equation:

$\boldsymbol{H}\boldsymbol{e}_{\boldsymbol{O}_{\boldsymbol{2}}}\boldsymbol{=}\boldsymbol{H}\boldsymbol{e}_{\boldsymbol{O}_{\boldsymbol{2}}}^{\boldsymbol{o}}\boldsymbol{\cdot}\boldsymbol{e}^{\boldsymbol{-}\boldsymbol{\alpha}\boldsymbol{(}\frac{\boldsymbol{1}}{\boldsymbol{T}}\boldsymbol{-}\frac{\boldsymbol{1}}{\boldsymbol{T}^{\boldsymbol{o}}}\boldsymbol{)}}$ Equation 5

$He_{O_{2}}^{o}$is the Henry Coefficient at standard conditions (in mol/m^3^/Pa), T is the reactor temperature (in K) and T^o^ is the standard temperature (298 K). The α parameter from Equation 5 is equal to ΔH/R (in K), where ΔH^o^ is the standard enthalpy of dissolution of the gas in water and R is the ideal gases constant (in Pa·m^3^/mol/K).

Substituting the parameters from the equations 4 and 5 for the values shown in the Table S 2, the value of $He_{O_{2}}$ was 1.56 x 10^-5^ mol/m^3^/Pa and the value of $C_{O_{L}}^{*}$ was equal to 0.425 mmol_O2_/L. Using the formula 5.3, it was determined that the required $k_{L}a$ are around 0.008 s^-1^.

Table S 2. Values of the parameters used to calculate the $\boldsymbol{k}_{\boldsymbol{L}}\boldsymbol{a}$ and the$\boldsymbol{C}_{\boldsymbol{O}_{\boldsymbol{L}}}^{\boldsymbol{*}}$ of oxygen in water (Sander 2015) in the conditions of the bioreactor.

| Parameter | Value [units] |
| --- | --- |
| $He_{O_{2}}^{o}$ | 1.3·10^-5^ mol/m^3^/Pa |
| $\alpha$ | 1400 K |
| $T$ | 310 K |
| $T^{o}$ | 298 K |
| $P_{O_{2}}$ | $\frac{20.98}{100}\cdot1.3 bar\cdot\frac{100000 Pa}{1 bar}=27274 Pa$* |

* The air contains 20.98% of O_2_ and the reactor is operated at an overpressure of +0.3 bar.

In order to determine the settings of the bioreactor, theinformation contained in **Figure S 2** was used (Karimi et al. 2013). **Figure S 2** shows the *kLa* values obtained from different combinations of stirring rate (rpm) and aeration rate (vvm). The values displayed in this graph are close to the values observed in previous experiments performed in our group using the same bioreactor than the one which was used for the present study. It was concluded that the aeration rate should be 0.55 vvm for both cases, while the stirring rate should be around 400 rpm. However, this theoretically calculated values were used as initial setting: the experimental conditions were expected to be different because some factors such as the viscosity and the presence of antifoam affects the volumetric oxygen transfer coefficient (*kLa*). Therefore, the stirring speed was adjusted during the continuous cultivation to achieve oxygen limitation.


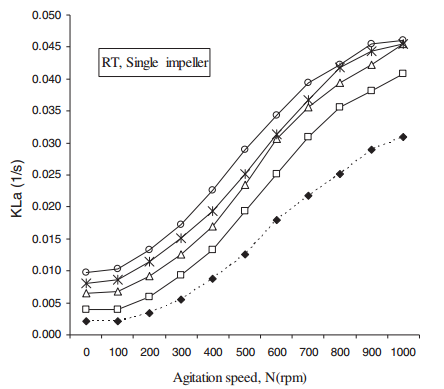


aaa


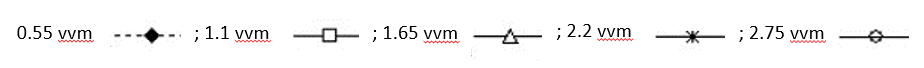


Figure S 2: Relation of the stirring rate, the aeration rate and the *kLa*.

Supplementary material 4: Setup and operation of the continuous cultures

In the continuous cultures, the medium was adjusted to the final pH of 7.0 inside the reactor. BC Antifoam 86/013 (Basildon Chemicals, United Kingdom) was employed to prevent excesive foaming. These cultures were performed in a 2 L vessel with a working volume of 1 L. The experimental conditions were controlled with a BIOSTAT B Plus Twin Bioreactor (Sartorius, Germany). The dilution rate was set to 0.05 h^-1^, the temperature was set to 37 °C and the pH was maintained at 7.0 using 2 M H_2_SO_4_ or 2 M KOH. The inflow pump was maintained constant while the outflow pump was controlled with a level sensor, keeping the volume of the culture at a constant value. Compressed air was injected in the reactor with a rate of 0.55 v.v.m. The reactor was operated at an over-pressure of 0.3 bar. The dissolved oxygen tension (DOT) was measured with a probe (Mettler-Toledo, Switzerland) and pH was measured with a probe (Applikon, The Netherlands). The stirring mechanism consisted of twin Rushton Turbine impellers and the stirring rate was the variable to be manipulated to achieve the desired oxygen-limiting conditions. The out-gas was cooled down using a condenser, and it was dried (permapure, Perma Pure LLC, NJ, USA) to avoid the interference of the gaseous water. The fractions of oxygen and CO_2_ in the dried out-gas were monitored online with a combined CO_2_ (infrared) and oxygen (paramagnetic) gas analyzer (NGA 2000, Rosemount Analytical, Germany).

For the determination of the actual dilution rate, the liquid leaving the reactor was disposed in a tank and the variation of the weight over time of this tank determined the rate of the liquid leaving the reactor. The actual dilution rate was calculated monitoring the flow-rate leaving the bioreactor during the steady state and the total volume of liquid inside the bioreactor, which was measured at the end of the fermentation.

Supplementary material 5: Analytical methods

For the determination of the biomass concentration by gravimetric analysis, triplicates samples of 5 ml of broth were taken from the bioreactor. The exact weight of the sample was determined by the difference in mass between the empty and filled collection tubes. The broth samples were washed and filtrated using pre-weighed Pall membrane filters (Sigma-Aldrich, Germany) with a pore size of 0.2 μm. The retained biomass samples were dried at 70 °C during 48 h and the filters were weighted again.

Samples in triplicate of cell-free medium were taken to analyse the concentration of organic compounds by HPLC. Broth samples of approximately 1 mL were placed in syringes and filtrated using glycerol-free 0.22 μm filters. For the quantification of glucose, ethanol, formate, lactate, succinate and acetate, HPLC with an Aminex HPX-87 H ion exclusion column (Bio-Rad, USA) was used. The mobile phase consisted of phosphoric acid 0.05 %, with a flow-rate of 0.6 ml/min. The concentration of pyruvate was quantified using the Pyruvic Acid Assay Kit (Megazyme, Ireland).

Sucrose was measured using a Dionex ICS – 5000 HPIC system with AS-AP sampler using the column Carbopac PA-20 3·150 mm column and Aminotrap 3·30 mm pre-column. The temperature of the column was maintained at 30 °C, the detector at 15 °C and the samples at 10 °C. The flow rate was always constant at 0.5 mL/min. The elution was performed applying a gradient which went from 0 % to 5 % of a NaOH 200 mM solution. This gradient increased at a constant rate for 15 minutes. Afterwards, the column was cleaned using a 20 % sodium acetate solution 0.5 M in 200 mM NaOH for 5 minutes. Finally, the column was re-equilibrated using ultra-pure water (resistivity 18.2 MΩ·cm at 25 °C) for 15 minutes. A volume of 10 μL of sample was used. Standard samples showed a linear range between 5 and 100 μM sucrose. Therefore, all samples were diluted to fit in this range.

Two samples of 20 mL of broth where taken to determine the total organic carbon (TOC) and total nitrogen (TN). One of the samples was directly stored at -20 °C (total broth sample), while the other sample was centrifuged at 4000 g (4 °C) for 15 minutes. The supernatant of this second sample was stored at -20 °C (filtrate sample). The TOC content in the filtrate and in the total broth samples was measured with a TOC analyser (Shimadzu TOC-L CSH, Japan), which was supplemented with the Nitrogen unit TNM-L (Shimadzu, Japan) for the quantification of the TN. The inorganic phosphate was measured in samples of filtered broth using the photometric assay LCK 350 Kit (Hach Lange, Danaher, U.S.A.). To quantify the glycogen content, broth samples of 8 mL were freeze-dried overnight. The pellets were transferred to a pre-weighted 2 mL tube. The weight of the sample was determined using a microscale. Ethanol 75 % (v/v) was pre-heated in a water bath at 95 °C. 1 mL of pre-heated ethanol was added to the samples, which were placed in a water bath at 95 °C for 3 minutes. With this step, the cytoplasmic membranes were dissolved and the intracellular content was liberated from the cells. After boiling the ethanol for 3 minutes, the samples were placed in a SpeedVac Concentrator (Thermo Fisher Scientific, U.S.A.) at 45 °C and a vacuum rate of 70 torr/min. The samples were taken from the SpeedVac when all the ethanol was evaporated. The resulting pellet was dissolved in 400 µL of buffer 0.25 M Na_2_CO_3_ pH 5.2. The pH of the buffer was adjusted using acetic acid 1 M. 100 µL of amyloglucosidase (Sigma-Aldrich, Germany) at a concentration of 6 U/mL, were added to the sample. The amyloglucosidase catalyses the hydrolysis of glycogen releasing monomers of glucose. The samples were placed in a thermomixer at 57 °C and 600 rpm using cycles of 30 seconds mixing and 15 seconds standing. The reaction was left overnight. The glucose concentration in every sample was measured in triplicates using a D-glucose quantification kit (Boehringer Mannheim, Germany).

## *q-rates calculation*

For the calculation of the biomass specific consumption/production (q-rates), the concentration of the by-products in the feeding flow is assumed to be zero. This assumption was consistent with the results of the quantification of the organic compounds by HPLC. The concentration of sucrose in the feeding flow was assumed to be the exactly 15 g/L after considering negligible the deviations during the preparation of 20 L of medium. Error propagation was considered to calculate the uncertainties associated with variables derived from more than one experimentally measured value.

*Enzymatic assays*

For the SucP and the phosphoglucomutase assays, MOPS 50 mM at pH 7 was used as buffer. For the fructose-1,6-bisphosphatase assay, Tris 50 mM, MgCl_2_ 5 mM, NaCl 5 mM, glycerol 5% (v/v) at pH 8 was initially used. However, a glycerol-free buffer was employed later because the presence of glycerol generated a strong background signal, probably attributable to some reaction(s) enabling the transference of reducing equivalents from glycerol to NADP^+^.

Samples of broth from the batch or the continuous cultures were centrifuged at 4000 g during 10 minutes at 4 °C. The pellets were washed once with the aforementioned buffers and the suspension was centrifuged again using the same conditions. The washed pellets were re-suspended in 10 mL of the corresponding buffer complemented with 2 mM D/L-Dithiothreitol and cOmplete Proteases Inhibition Cocktail™ (Roche, Switzerland) following the instructions of the manufacturer. The re-suspended pellets were sonicated on ice to minimize protein denaturation. Sonicated samples were centrifuged at 10000 g at 4 °C for 30 minutes to separate soluble proteins from cell debris. The obtained supernatants were considered cell-free extracts. Total protein content in the cell-free extracts was quantified using the method described by Bradford (Bradford 1976). Solutions of bovine serum albumin (Sigma-Aldrich, U.S.A.) at known concentrations were used as standards.

The specific SucP activity was measured using a coupled assay (Silverstein et al. 1967). The assay mixture was composed of 50 mM MOPS at a pH of 7, 0.5 mM NADP^+^, 8 mM of sucrose, 5 mM potassium phosphate (pH 7), 0.6 U/mL of glucose-6-phosphate dehydrogenase (Roche, Switzerland), 0.6 U/mL of phosphoglucomutase (Roche, Switzerland) and cell free extract. This way, the phosphorolysis of sucrose was stoichiometrically coupled to the production of NADPH. The production of NADPH was followed spectrophotometrically at 340 nm using a microplate reader Sinergy HTX (Biotek, USA). An assay similar to the one described for SucP was used to measure the specific phosphoglucomutase activity. However, in this case, the phosphoglucomutase was not added as an auxiliary enzyme.

The fructose-1,6-bisphosphatase specific activity was measured using a coupled assay (Rittmann et al. 2003). The assay mixture contained 50 mM Tris buffer at pH 8, 5 mM MgCl_2_, 5 mM NaCl, 0.5 mM NADP^+^, 0.4 mM of fructose-1,6-bisphosphate, 1.2 U/mL of glucose-6-phosphate dehydrogenase (Roche, Switzerland), 1.2 U/mL of phosphoglucoisomerase (Roche, Switzerland) and cell-free extract. The production of NADPH was followed spectrophotometrically at 340 nm.

Supplementary material 6: Non-reconciled rates observed in the continuous cultures of SUC-HYD and SUC-PHOSP under oxygen-limiting conditions.

| Specific rates (mmol/g_CDW_/h) | SUC-HYD | | | | | | | | | SUC-PHOSP | | |
| --- | --- | --- | --- | --- | --- | --- | --- | --- | --- | --- | --- | --- |
| Sucrose | -0.4427 | | ± | | 0.0448 | | | | -0.5139 | | ± | 0.0286 |
| O_2_ | -2.3653 | | ± | | 0.0543 | | | -3.3029 | | | ± | 0.1909 |
| CO_2_ | 2.4143 | | ± | | 0.035 | | | | | 3.3978 | ± | 0.1916 |
| Formate | b.d.l. | | | | | | | | | 0.0348 | ± | 0.0072 |
| Acetate | 0.0036 | | | ± | | 0.0002 | | | | 0.0066 | ± | 0.0004 |
| Lactate | 0.0225 | | | ± | | 0.0007 | | | | 0.0129 | ± | 0.0008 |
| Pyruvate | 0.0365 | | | ± | | 0.0076 | | | | 0.1111 | ± | 0.0187 |
| Succinate | 0.0752 | | | ± | | 0.0128 | | | | 0.0825 | ± | 0.0133 |
| Biomass concentration (g_CDW_/L) | 4.9737 | | | ± | | 0.0682 | | | | 3.9877 | ± | 0.2217 |
| Dilution rate (h^-1^) | 0.0503 | | | | | | | | | 0.0468 | | |
| Biomass yield (g_CDW_/g_Sucrose_) | 0.3321 | ± | | | | | 0.0336 | | | 0.266 | ± | 0.0148 |

b.d.l.: bellow the detection limit

Supplementary material 7. TOC/TN measurements. Carbon balance of the by-products, C fraction in biomass and N:C ratio in biomass

The TOC measurements of the filtrate indicate the non-biomass carbon present in the bioreactor. The balance between this value and all the detected components present in the media indicated that all the main by-products had been measured. The balances closed with a fraction above 90 % in both cases. These results are shown in the Table S 3.

Table S 3. Carbon balance of the TOC of the filtrate and the measured components of the mixture.

| Strain | Component | Measured concentration (mM) | Number of C | Carbon concentration (mM) | TOC filtrate (mM) | Carbon balance (Measur./TOC) |
| --- | --- | --- | --- | --- | --- | --- |
| SUC-HYD | Succinate | 7.43 ± 1.26 | 4 | 29.72 ± 5.04 | 51.087 | 95.49 ± 10.81 % |
|  | Lactate | 2.228 ± 0.065 | 3 | 6.683 ± 0.195 |  |  |
|  | Formate | 0 | 1 | 0 |  |  |
|  | Acetate | 0.356 ± 0.024 | 2 | 0.711 ± 0.048 |  |  |
|  | Pyruvate | 3.61 ± 0.75 | 3 | 10.830± 2.25 |  |  |
|  | Sucrose | 0.070 ± 0.007 | 12 | 0.838 ± 0.084 |  |  |
| SUC-PHOSP | Succinate | 7.034 ± 0.168 | 4 | 28.136 ± 4.272 | 69.39 | 92.44 ± 8.99 % |
|  | Lactate | 1.099 ± 0.038 | 3 | 3.298 ± 0.114 |  |  |
|  | Formate | 2.968 ± 0.588 | 1 | 2.967 ± 0.588 |  |  |
|  | Acetate | 0.559 ± 0.012 | 2 | 1.118 ± 0.024 |  |  |
|  | Pyruvate | 9.474 ± 1.502 | 3 | 28.422 ± 4.506 |  |  |
|  | Sucrose | 0.017 ± 0.001 | 12 | 0.202 ± 0.012 |  |  |

The carbon content of the biomass and the carbon to nitrogen ratio of the biomass were calculated using the comparison between the TOC and the TN values in the broth and in the filtrate. The difference between these two mixtures corresponds to the biomass TOC and the TN present in the biomass fraction. The results are shown in the Table S 4.

Table S 4: TOC and TN measurements of the samples taken from the chemostat cultivations of SUC-HYD and SUC-PHOSP

| Sample ID | TOC results (mCmol/L) | TN results (mNmol/L) |
| --- | --- | --- |
| Filtrate-SUC-HYD | 51.858 | 54.907 |
| Filtrate-SUC-HYD | 50.317 | 56.171 |
| Filtrate-SUC-PHOSP | 109.667 | 73.643 |
| Filtrate-SUC-PHOSP | 69.392 | 70.45 |
| Broth-SUC-HYD | 271.667 | 109.571 |
| Broth-SUC-HYD | 271.167 | 111.5 |
| Broth-SUC-PHOSP | 272.917 | 116.143 |
| Broth-SUC-PHOSP | 261 | 115.357 |

The calculations to obtain the N:C ratio and the carbon content of biomass are shown in the Table S5.

Table S 5. Carbon to nitrogen ratio in biomass and biomass carbon composition based on the TOC and TN measurements of the samples taken from the chemostat cultivations of SUC-HYD and SUC-PHOSP

| Strain | Carbon in biomass (mCmol/L) | Nitrogen in biomass (mNmol/L) | N/C in biomass  (mNmol/mCmol) | Biomass concentration (g_CDW_/L) | Carbon composition in biomass (mCmol/g_CDW_) |
| --- | --- | --- | --- | --- | --- |
| SUC-HYD | 220.33 | 55.0 | 0.250 | 4.974 | 44.3 |
| SUC-PHOSP | 177.43 | 43.704 | 0.247 | 3.989 | 44.4 |

Supplementary material 8. Codon usage analysis

Analysis of the codon usage of *sucP*


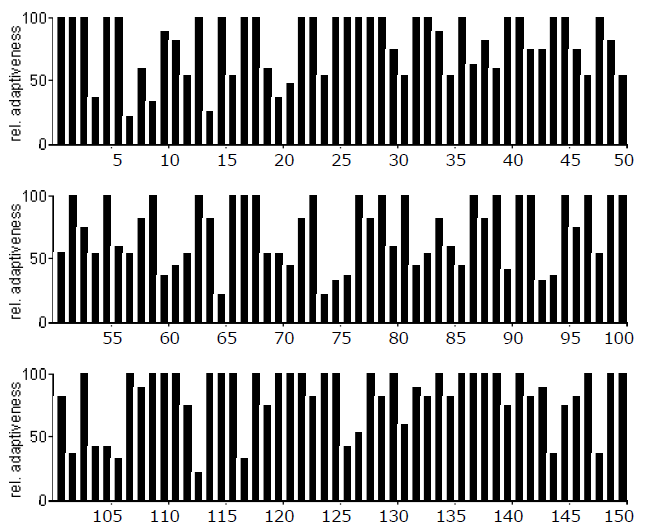

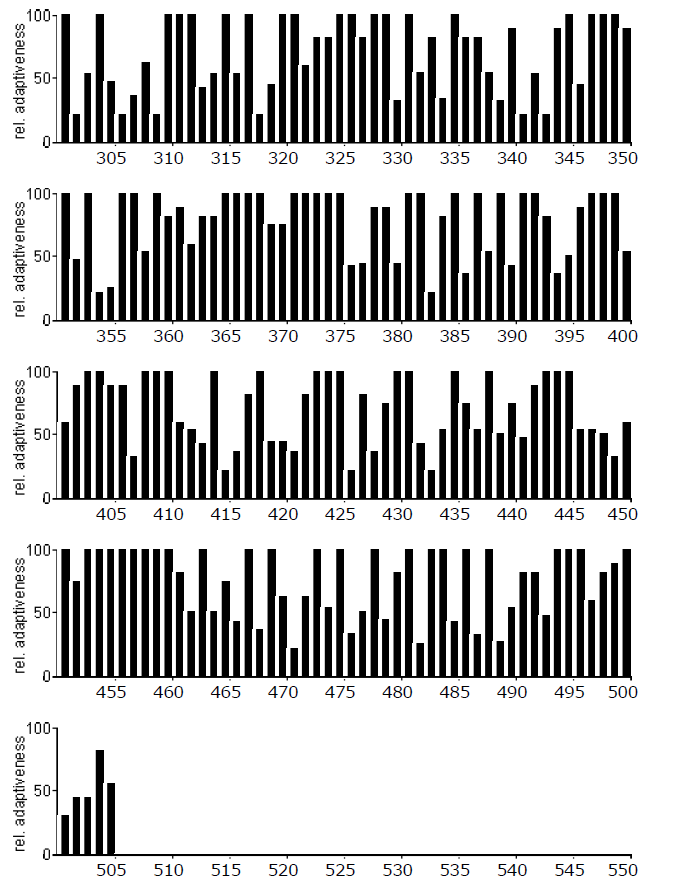


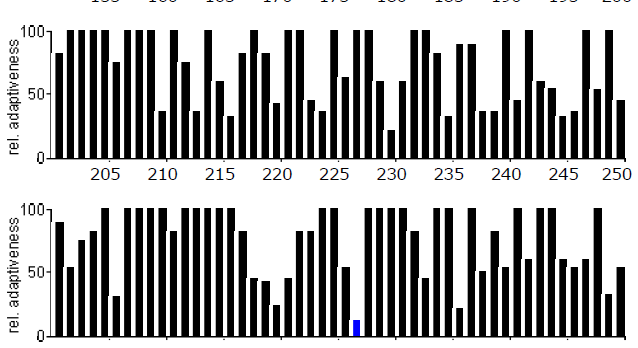
Analysis of the codon usage of *cscA*

Figure S 3: Codon bias of *sucP* compared to the codon usage of *E. coli* K-12. The codons with a codon usage lower than 20% are displayed in blue.


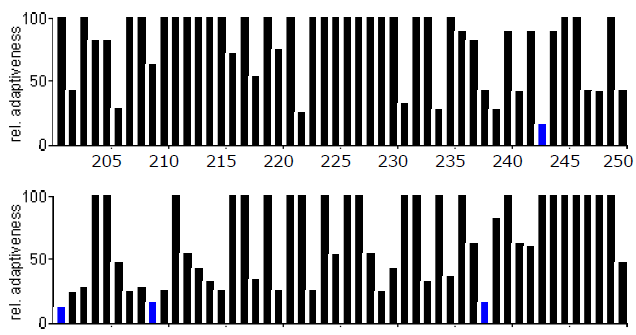

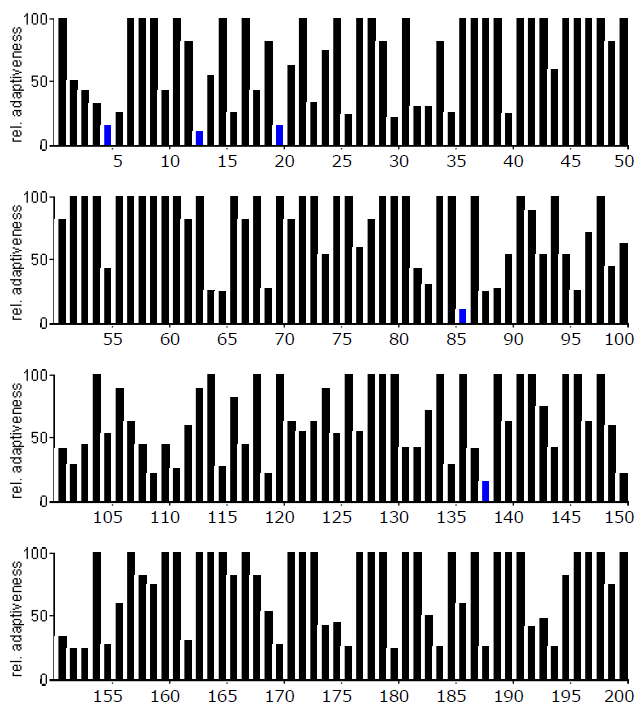


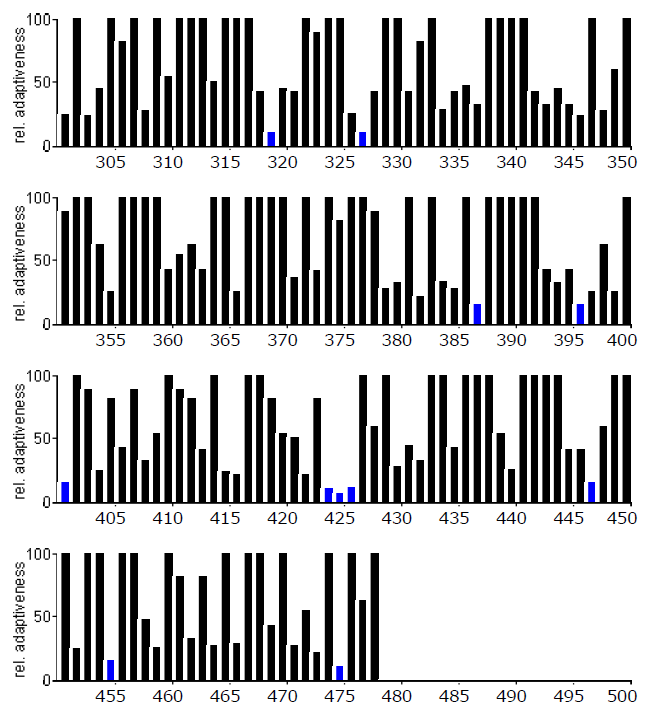

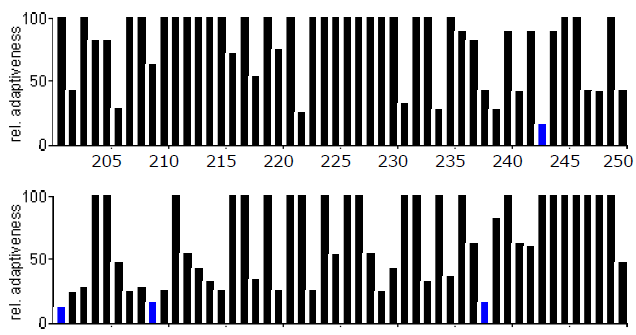


Figure S 4: Codon bias of *cscA* compared to the codon usage of *E. coli* K-12. The codons with a codon usage lower than 20% are displayed in blue.

Supplementary material 9. Overall carbon and electron balances

For the estimation of the biomass composition, the experimental data obtained (Table S 5) were compared with previous reference (Taymaz-Nikerel et al. 2010), yielding similar values. Moreover, the contribution of plasmid and protein burden were also considered (Supplementary material 10). Using the results of the q-rates, the overall carbon and electron balances were calculated. The results are shown in Table S 6, Table S 7, Table S 8 and Table S 9. The results show that the overall carbon and electron balances close, at least, at a 94%.

Table S 6. Carbon balance of the chemostat culture with SUC-HYD.

| Product | Specific consumption/production rate (mmol/g_CDW_/h) | Number of Carbons  (mCmol/mmol) | Total carbon consumed/produced (mCmol/g_CDW_/h) |
| --- | --- | --- | --- |
| Sucrose | - 0.443 ± 0.006 | 12 | - 5.312 ± 0.072 |
| O_2_ | - 2.196 ± 0.047 | 0 | 0 |
| CO_2_ | 2.211 ± 0.032 | 1 | 2.211 ± 0.032 |
| Formate | 0 | 1 | 0 |
| Acetate | 0.004 ± 0.0002 | 2 | 0.008 ± 0.0004 |
| Lactate | 0.023 ± 0.001 | 3 | 0.068 ± 0.002 |
| Pyruvate | 0.037 ± 0.008 | 3 | 0.110 ± 0.023 |
| Succinate | 0.064 ± 0.013 | 4 | 0.301 ± 0.051 |
| Biomass | 0.05 | 44.2* | 2.21 |
| Carbon balance ( $\frac{\mathbf{Total} \mathbf{carbon} \mathbf{consumed}\mathbf{-}\mathbf{Total} \mathbf{Carbon} \mathbf{produced}}{\boldsymbol{Total} \boldsymbol{Carbon} \boldsymbol{consumed}}\boldsymbol{\cdot}\boldsymbol{100}$) | | | 94.00 % ± 1.76% |

* The number of mmol of carbon per gram of CDW was taken from the Carbon content of the biomass from the TOC/TN measurements (Supplementary material 7).

Table S 7. Electron balance of the chemostat culture with SUC-HYD.

| Product | Specific consumption/production rate (mmol/g_CDW_/h) | Degree of reduction  (mmol e^-^/mCmol) | Total electrons consumed/produced (mmol e^-^/g_CDW_/h) |
| --- | --- | --- | --- |
| Sucrose | - 0.443 ± 0.006 | 48 | - 21.250 ± 0.288 |
| O_2_ | - 2.196 ± 0.047 | -4 | 8.7828 ± 0.189 |
| CO_2_ | 2.211 ± 0.032 | 0 | 0 |
| Formate | 0 | 4 | 0 |
| Acetate | 0.004 ± 0.0002 | 8 | 0.029 ± 0.002 |
| Lactate | 0.023 ± 0.001 | 12 | 0.270 ± 0.009 |
| Pyruvate | 0.037 ± 0.008 | 10 | 0.365 ± 0.076 |
| Succinate | 0.064 ± 0.013 | 14 | 1.053 ± 0.179 |
| Biomass | 0.05 | 198.9* | 9.94 |
| Electron balance ( $\frac{\mathbf{Total} \mathbf{electrons} \mathbf{consumed}\mathbf{-}\mathbf{Total} \mathbf{electorns} \mathbf{produced}}{\boldsymbol{Total} \boldsymbol{electrons} \boldsymbol{consumed}}\boldsymbol{\cdot}\boldsymbol{100}$) | | | 94.36 % ± 1.74 % |

* Based on the reference from Taymaz-Nikerel and co-workers (Taymaz-Nikerel et al. 2010) (4.5 mmol e-/mCmol).

Table S 8. Carbon balance of the chemostat culture with SUC-PHOSP.

| Product | Specific consumption/production rate (mmol/g_CDW_/h) | Number of Carbons  (mCmol/mmol) | Total carbon consumed/produced (mCmol/g_CDW_/h) |
| --- | --- | --- | --- |
| Sucrose | - 0.514 ± 0.028 | 12 | -6.167 ± 0.336 |
| O_2_ | - 3.012 ± 0.183 | 0 | 0 |
| CO_2_ | 3.113 ± 0.176 | 1 | 3.113 ± 0.176 |
| Formate | 0.035 ± 0.007 | 1 | 0.035 ± 0.007 |
| Acetate | 0.007 ± 0.0004 | 2 | 0.014 ± 0.001 |
| Lactate | 0.013 ± 0.001 | 3 | 0.0387 ± 0.003 |
| Pyruvate | 0.111 ± 0.019 | 3 | 0.333 ± 0.056 |
| Succinate | 0.083 ± 0.013 | 4 | 0.33 ± 0.053 |
| Biomass | 0.05 | 44.4* | 2.22 |
| Carbon balance ( $\frac{\mathbf{Total} \mathbf{carbon} \mathbf{consumed}\mathbf{-}\mathbf{Total} \mathbf{Carbon} \mathbf{produced}}{\boldsymbol{Total} \boldsymbol{Carbon} \boldsymbol{consumed}}\boldsymbol{\cdot}\boldsymbol{100}$) | | | 98.66 % ± 6.30 % |

* The number of mmol of carbon per gram of CDW was taken from the Carbon content of the biomass from the TOC/TN measurements (Supplementary material 7).

Table S 9. Electron balance of the chemostat culture with SUC-PHOSP.

| Product | Specific consumption/production rate (mmol/g_CDW_/h) | Degree of reduction  (mmol e^-^/mCmol) | Total electrons consumed/produced (mmol e^-^/g_CDW_/h) |
| --- | --- | --- | --- |
| Sucrose | - 0.514 ± 0.028 | 48 | -24.667 ± 1.344 |
| O_2_ | - 3.012 ± 0.183 | -4 | 12.050 ± 0.535 |
| CO_2_ | 3.113 ± 0.176 | 0 | 0 |
| Formate | 0.035 ± 0.007 | 4 | 0.139 ± 0.029 |
| Acetate | 0.007 ± 0.0004 | 8 | 0.056 ± 0.003 |
| Lactate | 0.013 ± 0.001 | 12 | 0.1548 ± 0.012 |
| Pyruvate | - 1. ± 0.019 | 10 | 1.110 ± 0.187 |
| Succinate | 0.083 ± 0.013 | 14 | 1.155 ± 0.186 |
| Biomass | 0.05 | 199.8* | 9.99 |
| Electron ( $\frac{\mathbf{Total} \mathbf{electrons} \mathbf{consumed}\mathbf{-}\mathbf{Total} \mathbf{electorns} \mathbf{produced}}{\boldsymbol{Total} \boldsymbol{electrons} \boldsymbol{consumed}}\boldsymbol{\cdot}\boldsymbol{100}$) | | | 96.68 % ± 6.16 % |

* Based on Taymaz-Nikerel and co-workers (Taymaz-Nikerel et al. 2010) (4.5 mmol e-/mCmol).

Supplementary material 10: Modifications in the *in-silico* model to consider plasmid and protein burden

Given the fact that the genes enabling the sucrose consumption were introduced in plasmids, some modifications were introduced in the *in-silico* model.

To calculate the contribution of the plasmids to the cellular weight, some assumptions were done. In *E. coli*, it has been previously shown that the cell volume decreases with the dilution rate following a second degree polynomial equation (Volkmer and Heinemann 2011). Therefore, the published data of plasmid weights at different dilution rates (Ataai and Shuler 1987) were fitted to a second degree polynomial equation to calculate the weight of the plasmids at 0.05 h^-1^. The result was 0.13 %. Because the reports about the change in the copy number are contradictory (Tal and Paulsson 2012), it was assumed that the number of plasmids per cell does not change with the dilution rate.

On the other hand, to calculate the contribution to the cellular weight of the heterologous proteins encoded in the plasmids pUC19-*cscAKB* or pUC19-*cscKB-sucP* further assumptions were made. Because it is known the DNA sequence of the plasmid-encoded genes, it was possible to calculate the molecular weight of the encoded proteins. Assuming that all the plasmid-encoded genes were expressed with similar strength (all them should be transcribed by the same RNA polymerase complex), if the contribution of one of the heterologous proteins is known, the contributions of the others can be calculated using the protein mass ratios. The contribution of the plasmid-encoded beta-lactamase (protein responsible for resistance to ampicillin) to the *E. coli* proteome (around 3 %) had been previously estimated (Ow et al. 2007). Based on that value, the contributions of the proteins encoded by the genes *cscB* (4.46 %), *cscK* (3.14 %), *cscA* (5.17 %) and *sucP* (5.34 %) to the proteome were calculated. Moreover, the specific sucrose phosphorylase activity measured in samples of SUC-PHOSP was employed to have an independent estimation of the abundance of the proteins. Using the K_Msucrose_ = 2.5 mM (Silverstein et al. 1967) and the k_cat_ = 200 s^-1^ (Cerdobbel et al. 2011) of the pure enzyme (obtained in conditions similar to our enzymatic assay), the specific activity (12.4 μmol/mg_soluble proteins_/min using 8 mM of sucrose) was used to calculate the relative abundance of the sucrose phosphorylase in the pool of soluble proteins. Because it is known that around 80 % of the *E. coli* proteome are soluble proteins (Almen et al. 2009) and proteins constitute 60-70 % of the cell dry weight (Taymaz-Nikerel et al. 2010), it was estimated that sucrose phosphorylase constitute around 4 % of the cell dry weight of SUC-PHOSP which is very similar to the 5 % calculated based on the mass ratio with the beta-lactamase (**see the supplementary file named Biomass composition**).

Knowing the contributions of the plasmids and the heterologous proteins to the cellular weight, and assuming that ashes represent 6.43 % of the cell dry weight (Taymaz-Nikerel et al. 2010), the contribution of the other biomolecules was re-scaled. The analyses of the amino acid composition of the heterologous proteins and the GC content of the plasmids showed that the relative atomic composition of these molecules do not diverge significantly from the typical *E. coli* protein or chromosomal DNA respectively. This way, the same relative atomic composition of the chromosomal DNA (C_1_H_1.05_N_0.385_O_0.616_P_0.103_) and the typical *E. coli* protein (C_1_H_1.58_N_0.288_O_0.309_S_0.009_) (Taymaz-Nikerel et al. 2010) were assigned for the plasmids and the heterologous proteins.

Overall, considering the contributions to the weight of the biomass and the relative atomic composition, the following proportion was estimated:

1 C-mol _lean biomass_ : 0.20421 mol _heterologous protein_ : 0.00126 mol _plasmid_.

All the calculations required to obtain this molar relationship can be found in the supplementary file named Biomass composition.

The relative elemental compositions calculated considering plasmid and recombinant protein burden were similar to the relative elemental composition obtained for the lean biomass (see supplementary file named Biomass composition). Moreover, the measurements of total nitrogen (TN) and total organic carbon (TOC) in the broth and the filtrate indicated that the ratios between nitrogen and carbon in the biomass remained similar in both strains, indicating no major differences in the biomass composition between them (Supplementary material 7). However, after the correction of the *in-silico* model to account plasmid and protein burden, the ATP associated with biomass formation increased compared to wild-type *E. coli*. The scripts to modify the biomass composition to account for the plasmid and protein burden are provided as supplementary files.

The movement of formate, acetate, lactate, pyruvate and succinate (organic acids) across the membrane was coupled with the movement of protons. However, considering that the concentration of organic acids in the reactor was lower than the levels that could trigger significant toxic effects (Luli and Strohl 1990; Warnecke and Gill 2005; Zaldivar et al. 1999), the ATP cost of eventual organic acid cycling across the membrane was neglected.

On the other hand, the ATP consumption associated with biomass formation was assumed to be the same for both strains for several reasons. First, both strains have the same background and they grew at the same conditions. Second, both strains contain plasmids of similar length, so the ATP expense associated to the plasmid burden should be similar (see estimations in the supplementary file named Biomass composition). Third, the TOC and TN measurements showed that the nitrogen to carbon ratio in biomass was not significantly different between the two strains, indicating that the protein content was similar. Another potential source of differences in the biomass-associated ATP expense between SUC-HYD and SUC-PHOSP could be the translation cost of the foreign proteins encoded in the plasmids. When considering these proteins, both strains have the same genes encoding for the sucrose/H^+^ symporter and the fructose kinase, differing only in the expression of a sucrose hydrolase (encoded by *cscA* from *E. coli* W) or a sucrose phosphorylase (encoded by *sucP* from *Bifidobacterium adolescentis)*. A high frequency of rare codons affects the folding of the protein, leading to larger amount of misfolded proteins (Rosano and Ceccarelli 2009) and consequently an increase in the ATP spending. To explore potential problems associated with the frequency of the codons present in the heterologous genes expressed in the constructed strains, the online tool Graphical Codon Usage Analyser was used (Fuhrmann et al. 2004). The analysis of the codon bias in the *cscA* and *sucP* sequences was performed employing the codon usage of *E. coli* K-12 as a reference. The results showed that for *cscA* only 20 codons out of 478 use a triplet with frequency below 20%. In the case of *sucP*, only one codon out of 505 had a codon usage below that threshold (Supplementary material 8). Therefore, eventual differences in the ATP expenses associated with the translation of rare codons were not considered.

Supplementary References

Almen MS, Nordstrom KJ, Fredriksson R, Schioth HB (2009) Mapping the human membrane proteome: a majority of the human membrane proteins can be classified according to function and evolutionary origin. BMC biology 7:50 doi:10.1186/1741-7007-7-50

Ataai MM, Shuler ML (1987) A mathematical model for prediction of plasmid copy number and genetic stability in *Escherichia coli*. Biotechnol Bioeng 30(3):389-97 doi:10.1002/bit.260300310

Bradford MM (1976) A rapid and sensitive method for the quantitation of microgram quantities of protein utilizing the principle of protein-dye binding. Anal Biochem 72:248-54

Cerdobbel A, De Winter K, Aerts D, Kuipers R, Joosten HJ, Soetaert W, Desmet T (2011) Increasing the thermostability of sucrose phosphorylase by a combination of sequence- and structure-based mutagenesis. Protein Eng Des Sel 24(11):829-34 doi:10.1093/protein/gzr042

Fuhrmann M, Hausherr A, Ferbitz L, Schödl T, Hegemann P (2004) Monitoring dynamic expression of nuclear genes in *Chlamydomonas reinhardtii* by using a synthetic luciferase reporter gene. Plant Molecular Biology 55:869-881 doi:10.1007/s11103-005-2150-1

Karimi A, Golbabaei F, Mehrnia MR, Neghab M, Mohammad K, Nikpey A, Pourmand MR (2013) Oxygen mass transfer in a stirred tank bioreactor using different impeller configurations for environmental purposes. Iranian journal of environmental health science & engineering 10(1):6 doi:10.1186/1735-2746-10-6

Luli GW, Strohl WR (1990) Comparison of growth, acetate production, and acetate inhibition of *Escherichia coli* strains in batch and fed-batch fermentations. Applied and Environmental Microbiology 56(4):1004-1011

Orth JD, Fleming RM, Palsson BO (2010) Reconstruction and Use of Microbial Metabolic Networks: the Core *Escherichia coli* Metabolic Model as an Educational Guide. EcoSal Plus 4(1) doi:10.1128/ecosalplus.10.2.1

Ow DS, Lee RM, Nissom PM, Philp R, Oh SK, Yap MG (2007) Inactivating FruR global regulator in plasmid-bearing *Escherichia coli* alters metabolic gene expression and improves growth rate. J Biotechnol 131(3):261-9 doi:10.1016/j.jbiotec.2007.07.508

Rittmann D, Schaffer S, Wendisch VF, Sahm H (2003) Fructose-1,6-bisphosphatase from *Corynebacterium glutamicum*: Expression and deletion of the fbp gene and biochemical characterization of the enzyme. Archives of Microbiology 180(4):285-292 doi:10.1007/s00203-003-0588-6

Rosano GL, Ceccarelli EA (2009) Rare codon content affects the solubility of recombinant proteins in a codon bias-adjusted *Escherichia coli* strain. Microbial cell factories 8(1):41 doi:10.1186/1475-2859-8-41

Sambrook J, Fritsch EF, Maniatis T (1983) Molecular Cloning: A Laboratory Manual. Cold Spring Harbor Laboratory Press, New York (USA), Cold Spring Harbor, NY, USA

Sander R (2015) Compilation of Henry's law constants (version 4.0) for water as solvent. Atmos Chem Phys 15(8):4399-4981 doi:10.5194/acp-15-4399-2015

Sauer U, Canonaco F, Heri S, Perrenoud A, Fischer E (2004) The soluble and membrane-bound transhydrogenases UdhA and PntAB have divergent functions in NADPH metabolism of *Escherichia coli*. Journal of Biological Chemistry 279(8):6613-6619 doi:10.1074/jbc.M311657200

Schellenberger J, Que R, Fleming RMT, Thiele I, Orth JD, Feist AM, Zielinski DC, Bordbar A, Lewis NE, Rahmanian S, Kang J, Hyduke DR, Palsson B (2011) Quantitative prediction of cellular metabolism with constraint-based models: the COBRA Toolbox v2.0. Nature protocols 6:1290-1307

Silverstein R, Voet J, Reed D, Abeles RH (1967) Purification and Mechanism of Action of Sucrose Phosphorylase *. The Journal of biological chemistry 242(6):1338-1346

Tal S, Paulsson J (2012) Evaluating quantitative methods for measuring plasmid copy numbers in single cells. Plasmid 67(2):167-73 doi:10.1016/j.plasmid.2012.01.004

Taymaz-Nikerel H, Borujeni AE, Verheijen PJ, Heijnen JJ, van Gulik WM (2010) Genome-derived minimal metabolic models for *Escherichia coli* MG1655 with estimated in vivo respiratory ATP stoichiometry. Biotechnol Bioeng 107(2):369-81 doi:10.1002/bit.22802

Volkmer B, Heinemann M (2011) Condition-dependent cell volume and concentration of *Escherichia coli* to facilitate data conversion for systems biology modeling. PLoS One 6(7):e23126 doi:10.1371/journal.pone.0023126

Warnecke T, Gill RT (2005) Organic acid toxicity, tolerance, and production in *Escherichia coli* biorefining applications. Microbial cell factories 4:25-25 doi:10.1186/1475-2859-4-25

Zaldivar J, Martinez A, Ingram LO (1999) Effect of Selected Aldehydes on the Growth and Fermentation of Ethanologeic *Escherichia coli*. Biotechnology and Bioengineering 65(1):24-33
